# Cathepsin K‐Activated Probe for Fluoro‐Photoacoustic Imaging of Early Osteolytic Metastasis

**DOI:** 10.1002/advs.202300217

**Published:** 2023-06-21

**Authors:** Zhuorun Song, Jia Miao, Minqian Miao, Baoliang Cheng, Shenhua Li, Yinghua Liu, Qingqing Miao, Qing Li, Mingyuan Gao

**Affiliations:** ^1^ Key Laboratory of Radiation Medicine and Protection School for Radiological and Interdisciplinary Sciences (RAD‐X) Collaborative Innovation Center of Radiation Medicine of Jiangsu Higher Education Institutions Soochow University Suzhou 215123 China

**Keywords:** cathepsin K activation, early‐stage diagnosis, fluoro‐photoacoustic probe, osteolytic metastasis

## Abstract

Precise detection of early osteolytic metastases is crucial for their treatment but remains challenging in the clinic because of the limited sensitivity and specificity of traditional imaging techniques. Although fluorescence imaging offers attractive features for the diagnosis of osteolytic metastases, it is hampered by limited penetration depth. To address this issue, a fluoro‐photoacoustic dual‐modality imaging probe comprising a near‐infrared dye caged by a cathepsin K (CTSK)‐cleavable peptide sequence on one side and functionalized with osteophilic alendronate through a polyethylene glycol linker on the other side is reported. Through systematic in vitro and in vivo experiments, it is demonstrated that in response to CTSK, the probe generated both near‐infrared fluorescent and photoacoustic signals from bone metastatic regions, thus offering a potential strategy for detecting deep‐seated early osteolytic metastases.

## Graphical abstract

A cathepsin K activatable probe (CTSK‐APPA) is designed and synthesized for fluoro‐photoacoustic imaging of early osteolytic metastasis. It generates fluorescence and photoacoustic signals in response to cathepsin K, a biomarker specially secreted by osteoclasts, to allow for the early diagnosis of the osteolytic metastasis.

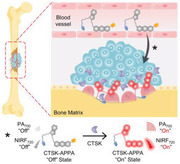

## 1 Introduction

Osteolytic metastases frequently occur in patients with advanced cancers, such as breast, lung, and renal cancers.^[^ ^1^ ^]^ Numerous studies have revealed that osteolytic metastases are caused by tumor cells that stimulate osteoclast (OC) activation and differentiation with the help of mature OCs^[^ ^2^ ^]^; meanwhile, osteoclastic bone damage triggers the liberation of growth factors, which further boosts the growth and metastasis of tumor cells to form an osteolytic vicious cycle.^[^ ^3^ ^]^ Malignant metastases lead to a series of skeletal‐related complications, including unbearable bone pain, pathological fractures, and hypercalcemia, severely reducing the quality of life and survival rate of patients.^[^ ^4^ ^]^ Early and precise diagnosis of osteolytic metastases is therefore crucial for achieving efficacious treatment but remains challenging because patients have no apparent symptoms during the early metastatic stage.^[^ ^5^ ^]^

Clinically, the diagnosis of bone metastases relies on conventional imaging techniques, including digital radiography (DR), computed tomography (CT), magnetic resonance imaging (MRI), and emission computed tomography, which encounter difficulties in detecting inconspicuous osteolytic lesions in bone metastases.^[^ ^6^ ^]^ Therefore, developing an innovative imaging technique with improved specificity and sensitivity is important for the early and precise diagnosis of osteolytic metastases.

Molecular optical imaging exhibits remarkable sensitivity and spatiotemporal resolution, whereas activatable molecular imaging probes enable the specific visualization of the subtle pathological changes owing to their improved signal‐to‐noise ratio.^[^ ^7^ ^]^ A number of activatable fluorescent probes have been developed for bone metastasis and OC imaging, but the limited penetration depth restricts their applications for deep‐tissue imaging.^[^ ^8^ ^]^ It is hopefully surmounted by photoacoustic (PA) imaging, which can detect ultrasonic signals from tissues up to 12 cm in depth. However, the sensitivity of PA imaging is not sufficiently high.^[^ ^9^ ^]^ In this context, innovative fluoro‐photoacoustic dual‐modality imaging that combines the complementary advantages of fluorescence and PA imaging techniques through a unique design of imaging probes is emerging as a powerful tool for imaging superficial tumors,^[^ ^10^ ^]^ atheromata,^[^ ^11^ ^]^ and acute organ injuries.^[^ ^12^ ^]^ Therefore, developing a fluoro‐photoacoustic imaging technique for diagnosing deep‐seated malignancies such as osteolytic metastases is interesting.

A key issue in constructing a reliable activatable probe is the selection of a relevant biomarker. Cathepsin K (CTSK), a cysteine protease, is specifically secreted by OCs to induce the degradation of the bone collagen matrix during bone resorption.^[^ ^2^, ^13^ ^]^ The essential role of CTSK in the initial stages of bone resorption makes it a promising target for the diagnosis of osteolytic lesions.^[^ ^14^ ^]^ In fact, only a few fluorescent probes for detecting CTSK in vivo have been reported.^[^ ^11^, ^15^ ^]^ However, the sensitivity and efficiency of these probes must be improved to meet the requirements for real‐time imaging of CTSK in the osteolytic metastatic microenvironment.

To address this issue, we report an activatable bone‐specific fluoro‐photoacoustic dual‐modality probe for ultrasensitive imaging of osteolytic metastasis in vivo. The most important feature differentiating the current probe from previous ones is that both NIR fluorescence and PA signals can be simultaneously unlocked by CTSK, which is expected to achieve more sensitive detection of CTSK in osteolytic metastasis in vivo.

## 2 Results and Discussion

### 2.1 Design and Synthesis of the CTSK‐APPA

**Figure 1:**
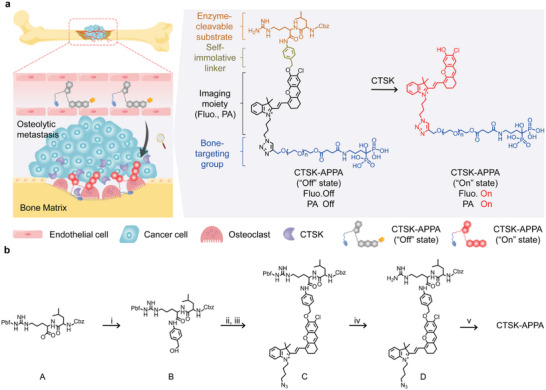
Design and synthesis of CTSK‐activatable fluoro‐photoacoustic probe. a) Schematic diagram showing the mechanism of CTSK‐APPA for imaging osteolytic metastasis. b) Synthetic route for CTSK‐APPA. Reagents and reaction conditions: i) p‐amino‐benzyl alcohol, 2‐(1H‐benzotriazole‐1‐yl)‐1,1,3,3‐tetramethyluronium hexafluorophosphate, hydroxy‐benzotriazole, N,N‐diisopropylethylamine (DIPEA), N,N‐dimethylformamide, 25 °C/4 h; ii) phosphorus tribromide, anhydrous tetrahydrofuran, 0 °C/3 h; iii) CyN_3_OH, DIPEA, anhydrous acetonitrile, 60 °C/4 h; iv) trifluoroacetic acid/dichloromethane (V/V) = 1:4, 0 °C/12 h; v) PEG‐ALN, copper (II) sulfate pentahydrate, sodium ascorbate, dimethyl sulfoxide/H_2_O (V/V) = 1:1, 25 °C/12 h.

**Figure** **1**a shows the molecular structure of the CTSK‐activated fluoro‐photoacoustic probe comprising four major components: 1) a hemicyanine dye (CyN_3_OH) simultaneously serving as a NIR fluorescent and PA signal moiety,^[^ ^10^, ^16^ ^]^ 2) a bone‐targeting alendronate group coupled to the central dye through a polyethylene glycol (2 kDa) (PEG2000) linker to improve the aqueous solubility of the whole probe, 3) a self‐immolative linker, p‐aminobenzyl alcohol (PABA), on the other side of the central dye, and 4) a CTSK‐specific peptide sequence carbobenzyloxy‐Leu‐Arg‐OH (Cbz‐Leu‐Arg‐OH) at a further distance.^[^ ^17^ ^]^ As the hydroxyl group of CyN_3_OH is caged by a self‐immolative PABA linker, the electron‐donating ability of the oxygen atom is significantly diminished. Therefore, the probe in its initial intact form is non‐fluorescent. However, when CTSK is present, the peptide segment is cleaved, which subsequently leads to the spontaneous 1,6‐elimination of the PABA moiety. Consequently, the probe becomes fluorescent. Meanwhile, the absorbance in the NIR region varies substantially, giving rise to an activated photoacoustic signal upon excitation at carefully selected positions.

**Figure 2:**
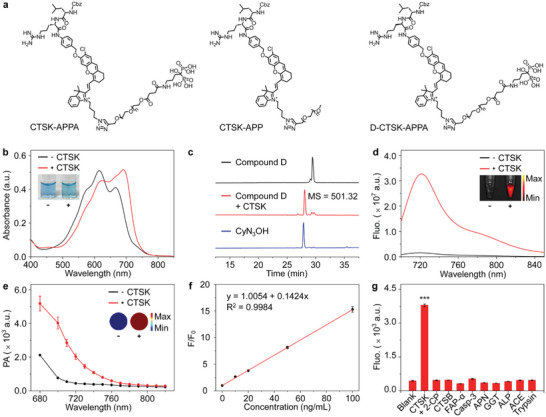
The activation performance of CTSK‐APPA in vitro. a) Chemical structures of CTSK‐APPA, CTSK‐APP, and D‐CTSK‐APPA. UV–vis absorption (b), HPLC analyses (c), fluorescence (Ex = 690 nm) (d), and wavelength dependent PA signal (Ex = 700 nm) (e) of CTSK‐APPA or compound D in the absence (−) or presence (+) of CTSK in MES buffer (50 mm, pH = 6.0, 37 °C/1 h) containing 0.1% DMSO (Insets: bright field photographs, fluorescence, and PA images of CTSK‐APPA in Eppendorf tubes recorded before or after treatment with CTSK). f) Fitted calibration curve of fluorescence enhancement (F/F_0_) of CTSK‐APPA in the presence of different concentrations of CTSK. g) Quantified fluorescence (Ex = 690 nm) at 720 nm of CTSK‐APPA activated by CTSK in the absence or presence of CTSK inhibitor *α*‐cyanopyridine (2‐CP) or other types of proteases as controls (37 °C/1 h), respectively. All values are shown as the means ± standard deviations (SD) (*n* = 3, one‐way ANOVA, ***p < 0.001).

The resulting cathepsin K‐activatable probe with PEG‐linked alendronate is denoted as CTSK‐APPA, and its synthetic route is shown in Figure 1b. First, the CTSK‐responsive peptide Cbz‐Leu‐Arg‐OH was prepared by typical solid‐phase peptide synthesis and was then conjugated to PABA to yield compound B. Compound B was then reacted with phosphorus tribromide to form an intermediate product that was further coupled with CyN_3_OH to form compound C. Upon deprotection with trifluoroacetic acid, compound C was converted into compound D, which was then conjugated to alendronate using the PEG2000 linker to yield the final CTSK‐APPA probe. In parallel, a probe bearing no alendronate group (denoted as CTSK‐APP) and a probe in which L‐arginine in the CTSK‐specific peptide sequence was replaced with D‐arginine (denoted as D‐CTSK‐APPA) were also synthesized as controls (**Figure** **2**a). A detailed characterization of all the compounds is provided in the Supporting Information.

### 2.2 In Vitro Studies

To investigate the responsiveness of the probes toward CTSK, their UV–vis absorption, NIR fluorescence, and PA performance were investigated. As shown in Figure 2b, CTSK‐APPA exhibited at least three absorption bands between 500 and 700 nm, with the strongest band located at 615 nm. After incubation with CTSK at 37 °C for 1 h, the strongest absorption decreased, and a new absorption band appeared at 690 nm. The same changes in the absorption spectra were also observed when incubating compound D with CTSK under the same conditions, suggesting that compound D, the major part of CTSK‐APPA, can be effectively cleaved by CTSK, followed by the elimination of PABA to expose the free hemicyanine segment CyN_3_OH, as supported by high‐performance liquid chromatography (HPLC) and mass spectrometry (MS) data (Figure 2c). Accompanied by the structural changes indicated by absorption spectroscopy, a strong fluorescence peak at 720 nm was observed, as shown in Figure 2d, with an on/off ratio close to 20.9, and an enhanced fluorescent quantum yield of 3.5% (Table S1, Supporting Information). The newly formed absorption band at 690 nm offered an enhancement of 5.2‐fold to the photoacoustic signal if irradiated at 700 nm (Figure 2e). Under identical conditions, CTSK‐APP exhibited comparable variations in all the aforementioned optical properties (Figure S1, Supporting Information). In contrast, D‐CTSK‐APPA containing D‐arginine at the cleavage site showed no obvious enhancement of fluorescence or PA signals (Figure S2, Supporting Information), indicating the nonresponsiveness of D‐CTSK‐APPA toward CTSK. Temporal variations in absorption and fluorescence were also investigated. As shown in Figure S3, Supporting Information, both the absorption at 690 nm and fluorescence at 720 nm gradually increased over the incubation period and reached corresponding plateaus after ≈60 min. According to the linear correlation between the fluorescence intensity and concentration of CTSK shown in Figure 2f, the detection limit for CTSK was extracted to be 0.58 ng mL^−1^, which is much lower than the best result reported in the literature (Table S2, Supporting Information).^[^ ^15^ ^]^

The responsiveness of CTSK‐APPA to CTSK was investigated using enzymatic kinetics studies. According to the results shown in Figure S4, Supporting Information, the enzymatic Michaelis–Menten constant (*K*_m_) and catalytic rate constant (*k*_cat_) for the CTSK‐specific activation of CTSK‐APPA were calculated to be 22.9 µM and 20.4 s^−1^, respectively. Then, a catalytic efficiency (*k*_cat_/*K*_m_) of 8.9 × 10^5^ M^−1^s^−1^ was derived, which is one order of magnitude higher than that of previously reported CTSK‐activatable fluorescent probes (Table S2, Supporting Information).^[^ ^15^ ^]^ To assess the specificity of CTSK‐APPA toward CTSK, the fluorescence of CTSK‐APPA was recorded and compared after it was incubated with CTSK in the presence of CTSK inhibitor *α*‐cyanopyridine (2‐CP) or with other proteases such as cathepsin B (CTSB), fibroblast activation protein‐*α* (FAP‐*α*), caspase‐3 (Casp‐3), aminopeptidase N (APN), glutamyltransferase (GGT), alkaline phosphatase (ALP), angiotensin converting enzyme (ACE), and trypsin, respectively. As shown in Figure 2g, almost no fluorescence activation was observed in the control group, demonstrating the outstanding specificity of CTSK‐APPA toward CTSK.

### 2.3 Bone Targeting and Activation Capacity

**Figure 3:**
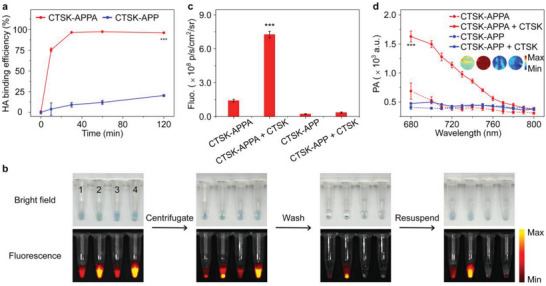
Bone targeting and activation capacity of probes. a) Hydroxyapatite (HA)‐binding efficiency of CTSK‐APPA and CTSK‐APP as a function of different incubation time points. b) The process for studying activation performance of CTSK‐APPA adsorbed by HA. The HA suspensions were pretreated with CTSK‐APPA or CTSK‐APP for 30 min and then incubated with or without CTSK for 1 h in MES buffer (50 mm, pH = 6.0) containing 0.1% DMSO. These treated suspensions were further centrifugated, washed, and resuspended. The bright field photographs and fluorescence images of the HA suspensions with (1) CTSK‐APPA, (2) CTSK‐APPA + CTSK, (3) CTSK‐APP, and (4) CTSK‐APP + CTSK. Fluorescence intensities (Ex = 660 nm) (c) at 720 nm and PA signals (Ex = 700 nm) (d) were recorded after CTSK‐APPA and CTSK‐APP adsorbed by HA were activated under different conditions. All values are shown as the means ± SD (*n* = 3, one‐way ANOVA, ***p < 0.001).

The bone‐binding affinity of CTSK‐APPA in vitro was characterized using a hydroxyapatite (HA) adsorption assay.^[^ ^18^ ^]^ As shown in **Figure** **3**a, the HA‐binding efficiency of CTSK‐APPA increased rapidly during the first 10 min of incubation and then stabilized after 30 min at approximately 96%. In contrast, CTSK‐APP exhibited poor HA binding affinity, with an efficiency close to 10% after 30 min of incubation. The 20% HA‐binding efficiency of CTSK‐APP after 2 h of incubation can be attributed to the hydrogen bonding interaction between the amino groups in CTSK‐APP and the hydroxyl groups on the surface of hydroxyapatite.

The activation performance of CTSK‐APPA adsorbed with HA was evaluated. After HA adsorbed on CTSK‐APPA was incubated with CTSK for 30 min, an intense fluorescence was observed (Figure 3b). Further quantitative analysis revealed that the on/off ratio for the fluorescence was 5.1 and the enhancement factor for the PA signal was approximately 2.8, as shown in Figure 3c,d. In contrast, the limited amount of CTSK‐APP adsorbed onto HA gave rise to negligible fluorescence and photoacoustic signal enhancement, as expected.

### 2.4 NIR Fluorescence Imaging of Bone Resorption Model and Cell Selectivity Analysis

**Figure 4:**
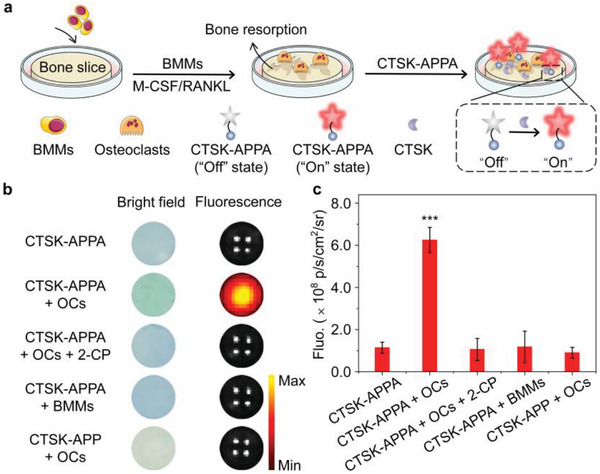
Preparation and imaging of bone resorption model. a) Schematic diagram for showing the detection of osteoclasts (OCs) in vitro on a bone resorption model with CTSK‐APPA through fluorescence imaging (b) and quantified fluorescence (Ex = 660 nm) (c) at 710 nm. All values are shown as the means ± SD (*n* = 3, one‐way ANOVA, ***p < 0.001).

The excellent cytocompatibility of the activatable probes (Figure S5, Supporting Information) encouraged the following subsequent in vitro detection of CTSK‐overexpressing OCs in a bone resorption model constructed using recombinant murine receptor activator of nuclear factor‐*κ*B ligand (RANKL) and macrophage colony‐stimulating factor (M‐CSF) to induce bone marrow‐derived macrophages (BMMs) to form OCs (**Figure** **4**a and Figure S6, Supporting Information). As shown in Figure 4b,c, the bone‐resorption slice exhibited a stronger NIR fluorescence signal after incubation with CTSK‐APPA than the normal bone slices treated with CTSK‐APPA and BMMs/CTSK‐APPA, respectively, owing to the absence of OCs. In addition, the bone‐resorption slice presented remarkably suppressed NIR fluorescence when pretreated with 2‐CP, further supporting the excellent specificity of CTSK‐APPA toward CTSK‐overexpressing OCs. In contrast, CTSK‐APP failed to produce a fluorescence signal from CTSK‐overexpressing OCs, suggesting that the alendronate moiety was essential for binding the activatable probe to bone tissues.

To further confirm the specificity of CTSK‐APPA toward CTSK‐overexpressing OCs, cell selectivity experiments were performed by incubating the probes with different cells, including OCs, BMMs, MDA‐MB‐231 cells, MCF‐7 cells, and neutrophils.^[^ ^19^ ^]^ As shown in Figure S7, Supporting Information, the OCs showed strong NIR fluorescence signals after incubation with CTSK‐APPA, which could be effectively reduced by 2‐CP. In striking contrast, other cells did not induce NIR fluorescence activation of CTSK‐APPA, and D‐CTSK‐APPA failed to be activated by OCs, validating the high selectivity of CTSK‐APPA toward CTSK‐overexpressing OCs.

### 2.5 Biocompatibility and Metabolic Pathway

It is important to learn about the bone marrow toxicity and metabolic pathways of a given bone‐targeting biomaterial before further in vivo applications, but these are easily ignored. To this end, blood and urine samples were collected after CTSK‐APPA was intravenously injected into healthy living mice. Blood samples were subjected to routine examinations to determine the hemogram index of red blood cell (RBC) and white blood cell (WBC) counts. No apparent differences were observed between the CTSK‐APPA and saline groups, as shown in Figure S8, Supporting Information, indicating that CTSK‐APPA had excellent biocompatibility. Further urine analysis indicated that the renal clearance efficiency of CTSK‐APPA reached 58.4% 24 h post‐injection (Figure S9, Supporting Information), which was close to that of CTSK‐APP (59.7%). This good renal clearance ability could be attributed to the excellent blood residence behavior of PEG2000, which was further demonstrated through organ distribution studies (Figure S10, Supporting Information).

### 2.6 NIR Fluorescence and PA Imaging of Osteolytic Metastasis

**Figure 5:**
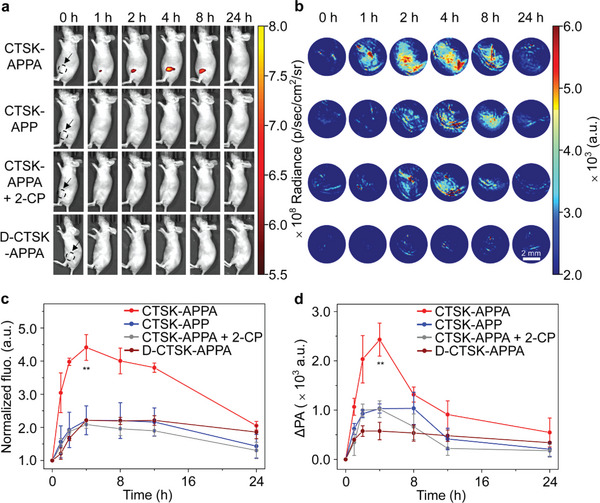
NIR fluorescence and PA imaging of the osteolytic metastasis in vivo. Representative NIR fluorescence (a) and PA images (b) of mice with MDA‐MB‐231/Luc‐induced osteolytic metastasis, recorded 24 h post intravenous injection of CTSK‐APPA, CTSK‐APP, 2‐CP followed by CTSK‐APPA, and D‐CTSK‐APPA, together with the temporal NIR fluorescence (Ex = 660 nm) at 720 nm (c) and PA signals (Ex = 700 nm) (d) of the region of interest. All values are shown as the means ± SD (*n* = 3, one‐way ANOVA, **p < 0.01).

Based on these outstanding results, we attempted to determine the potential of CTSK‐APPA to detect osteolytic metastases in vivo. An MDA‐MB‐231/Luc‐induced osteolytic metastatic mouse model was constructed (Figure S11a,b, Supporting Information).^[^ ^20^ ^]^ Figure S11c–e, Supporting Information, shows that conventional DR, CT, and MRI failed to detect the small osteolytic metastases that exhibited NIR fluorescence and PA signals after CTSK‐APPA was intravenously delivered (**Figure** **5**a,b). Further quantitative analyses revealed that the intensities of both these signals increased over time to reach the corresponding maxima at 4 h post‐injection, followed by a decline in signal intensity, as shown in Figure 5c,d. The control probe, CTSK‐APP, presented similar signal variations; however, the signal intensities were much lower, demonstrating the rational choice of alendronate as a bone‐targeting moiety. Unsurprisingly, D‐CTSK‐APPA also exhibited lower NIR fluorescence and PA signals than CTSK‐APPA due to its lack of CTSK‐activation ability. Moreover, the NIR fluorescence and PA signals of CTSK‐APPA were reduced to the CTSK‐APP levels when the CTSK inhibitor 2‐CP was preinjected, demonstrating the specificity of the dual‐modality probe for osteolytic metastasis imaging in vivo. As shown in Figure S12, Supporting Information, more supportive evidence was obtained from ex vivo NIR fluorescence imaging at 4 h post‐injection. In addition, histological studies showed no histological changes after the injection of CTSK‐APPA, indicating its biocompatibility (Figure S13, Supporting Information).

## 3 Conclusion

In conclusion, we developed a bone‐targeted imaging probe for early osteolytic metastasis imaging using NIR fluorescence and PA signals simultaneously activated by OC‐secreted cathepsin K. The dual‐modality imaging probe comprises the NIR fluorescent hemicyanine dye that is linked to the CTSK‐cleavable peptide substrate through a self‐immolative linker on one side and a bone‐targeting alendronate group through the PEG2000 linker on the other side. Systematic in vitro studies revealed that the CTSK‐APPA probe not only has a strong bone‐binding affinity but also a specific and sensitive response to CTSK to exhibit NIR fluorescence and PA signals. Owing to these prominent properties, CTSK‐APPA can specifically delineate the osteolytic metastases in live mice through NIR fluorescence and PA dual‐modality imaging after intravenous delivery, which is difficult to achieve using conventional imaging techniques. Although bioluminescence imaging shows a better imaging performance than fluorescence and PA imaging techniques, it requires engineered luciferase expression in the investigated system, which limits flexibility and increases costs. In contrast, fluoro‐photoacoustic dual‐modality imaging that can integrate their respective merits via the rational design of molecular probes is more flexible and cheaper, thus showing great potential in clinical applications. To the best of our knowledge, the current study provides the first activatable fluoro‐photoacoustic probe for osteolytic metastasis detection. The probe design strategy holds significant promise for creating innovative imaging probes for other types of diseases.

## 4 Experimental Section

For detailed experiment information, please refer to Supporting Information.

### Animals

All experiments with specific pathogen‐free (SPF) grade 4‐week‐old BALB/c nude mice were carried out in accordance with the guidelines approved by the Ethics Committee of Soochow University (Suzhou, China) (approval number: 202112A0295).

### Statistical Analysis

Fluorescence and PA data were processed by ROI analysis by using Living Image software (version 4.0) and ViewMSOT, respectively. Normalized in vivo fluorescence data were obtained by dividing the data at different time points by the ROI values at 0 h. The data are expressed as the means ± SD of at least triplicate samples (*n* = 3) unless stated otherwise. Statistical comparisons were performed using *t*‐test for two groups and one‐way analysis of variance (ANOVA) for more than two groups: n.s., no significant difference, *p < 0.05, **p < 0.01, ***p < 0.001. For all tests, statistical analyses were performed using OriginPro (version 8.5), and a two‐sided *p* < 0.05 was deemed statistically significant.

## Conflict of Interest

The authors declare no conflict of interest.

## Supporting information

Supporting Information

## Data Availability

The data that support the findings of this study are available from the corresponding author upon reasonable request.
