# Supplementary material for: Cathepsin K‐Activated Probe for Fluoro‐Photoacoustic Imaging of Early Osteolytic Metastasis
Source: Adv Sci (Weinh). 2023 Jun 21;10(24):2300217. doi: 10.1002/advs.202300217 (PMC10460880; doi:10.1002/advs.202300217)
Supplement: Supplementary file 1 — Supporting Information [file ADVS-10-2300217-s001.pdf]

ADVANCED SCIENCE
Open Access

Supporting Information

for *Adv. Sci.*, DOI 10.1002/advs.202300217

Cathepsin K-Activated Probe for Fluoro-Photoacoustic Imaging of Early Osteolytic Metastasis

Zhuorun Song, Jia Miao, Minqian Miao, Baoliang Cheng, Shenhua Li, Yinghua Liu, Qingqing Miao, Qing Li and Mingyuan Gao**

Supporting Information

Cathepsin K-Activated Probe for Fluoro-Photoacoustic Imaging of Early Osteolytic Metastasis

Z. Song, J. Miao, M. Miao, B. Cheng, S. Li, Y. Liu, Q. Miao, Q. Li, M. Gao

Experimental Procedures

Chemicals. All chemicals used in the experiments were purchased from Sigma-Aldrich unless otherwise described. Commercially available reagents were used without further purification unless noted otherwise. Fmoc-Arg-OH, Z-Leu-OH, 2-(1H-benzotriazole-1-yl)-1,1,3,3-tetramethyluronium hexafluorophosphate (HBTU), hydroxybenzotriazole (HOBT), 2-Chlorotrityl chloride polystyrene were purchased from GL Biochem (Shanghai) for solid phase peptide synthesis (SPPS). Recombinant human cathepsin B (CTSB), fibroblast activation protein-α (FAP-α), caspase-3 (Casp-3), alkaline phosphatase (ALP) and aminopeptidase N (APN) were purchased from R&D Systems. Angiotensin converting enzyme (ACE) was purchased from Solarbio and recombinant trypsin was purchased from Beyotime Biotechnology. Dulbecco's modified eagle medium (DMEM), Leibovitz's L-15 (L-15), Minimum Essential Medium α (MEM α), fetal bovine serum (FBS), Trypsin-EDTA (0.05%) and penicillin-streptomycin (PS) were purchased from Gibco. Recombinant murine receptor activator of nuclear factor-κB ligand (RANKL) and macrophage colony-stimulating factor (M-CSF) were purchased from Peprotech. Water was supplied by Milli-Q Plus System (Millipore Corporation, Breford, USA).

Materials characterization. High-performance liquid chromatography (HPLC) analyses were performed on the Waters Alliance E2695 system equipped with an E2695 separation module, 2489 UV detector, and a Water XBridge C18 (4.6 × 250 mm) column with CH_3OH (0.1% of TFA) and water (0.1% of TFA) as the eluent. HPLC purification was performed on an Elite P3500 gradient preparative system equipped with a P3500 pump, UV3100 detector, and a CST Daiso C18 (20 × 250 mm,) column with CH_3OH (0.1% of TFA) or CH_3CN (0.1% of TFA) and water (0.1% of TFA) as the eluent. UV-Vis and fluorescence spectra were recorded on a PerkinElmer Lambda 35 and an Edinburgh FLS980 spectrofluorometer, respectively. Nuclear magnetic resonance spectra (^1H NMR) were obtained on a Bruker Avance 400 MHZ NMR. The matrix-assisted laser desorption/ionization time-of-flight mass spectrometry (MALDI-TOF-MS) spectra were recorded on an Ultraflextreme (Bruker). Confocal fluorescence imaging was performed with a FV1200 confocal microscopy (Olympus). Bioluminescence (BL), digital

radiography (DR), and fluorescence imaging were carried out by using an IVIS Spectrum imaging system (PerkinElmer). Photoacoustic (PA) imaging was obtained on a multispectral optoacoustic tomography (MSOT) scanner (iThera Medical). Computed tomography (CT) imaging was recorded on single-photon emission computed tomography imaging system (Olympus). Magnetic resonance (MR) imaging was recorded on a preclinical magnetic resonance solution system (MR solutions).

***In vitro* analysis of cathepsin K (CTSK) activation, selectivity, and sensitivity studies.** Probe CTSK-APPA, D-CTSK-APPA, or CTSK-APP (10 μM) was incubated with CTSK in MES buffer (50 mM, pH 6.0) containing 0.1% DMSO, 2 mM ethylene diamine tetraacetic acid (EDTA), 4 mM dithiothreitol (DTT) and 0.15% chondroitin sulfate (CS) at 37 °C for 1 h. After incubation at the indicated time, UV-Vis spectra, fluorescence spectra, fluorescence images, and PA images of the solutions were recorded. Probe CyP (10 μM) incubated with CTSK (200 ng/mL) for 1 h was further analyzed by HPLC. For selectivity study, CTSK-APPA (10 μM) in a 96-well plate was incubated with CTSK (100 ng/mL), CTSB (100 ng/mL), FAP-α (9×10^{-4} U/mL), Caspase-3 (100 ng/mL), APN (100 ng/mL), GGT (0.1 U/mL), ALP (0.1 U/mL), ACE (1×10^{-3} U/mL), and trypsin (10 U/L) at 37 °C for 1 h. For the inhibitory experiment, CTSK (100 ng/mL) was treated with CTSK inhibitor 2-Cyanopyrimidine (2-CP, 50 mM) at 37°C for 30 min. Then, the mixture was incubated with CTSK-APPA (10 μM) in MES buffer containing 0.1% DMSO, 2 mM EDTA, 4 mM DTT, and 0.15% chondroitin sulfate at 37°C for 1h. For the sensitivity study, CTSK-APPA (10 μM) was incubated with various concentrations of CTSK (0, 10, 20, 50, 100 ng/mL) at 37°C for 5 min. The limit of detection was determined from emission intensities according to the equation: $3\delta/k$, where δ represents the standard deviation of blank, and k represents the slope of the fitting plot of emission intensities. Fluorescence images were captured by using the IVIS Spectrum imaging system with excitation at 660 ± 10 nm and emission at 710 ± 10 nm. PA images were captured by using a MSOT scanner with excitation at 700 nm.

Kinetic assay. Different concentrations of CTSK-APPA or CTSK-APP (1, 2, 5, 10, 20, or 50 μM) were incubated with CTSK (100 ng/mL) in MES buffer containing 0.1% DMSO, 2mM EDTA, 4mM DTT, and 0.15% CS at 37 °C for 5 min. Fluorescence intensities of solution were measured for quantification analyses by a microplate reader (BioTek, Synergy 2) with excitation at 690 nm and emission at 720 nm. The kinetic parameters were calculated according to the Michaelis-Menten equation shown below: $V = V_{max}\times[S]/(K_m + [S])$, where V is initial velocity, and [S] is substrate concentration.

Determination of the fluorescence quantum yield and molar extinction coefficient. Fluorescence quantum yield (Φ_f) was determined by using ICG (Φ_f = 0.13 in DMSO) as a fluorescence standard. The quantum yield was calculated using the following equation: $\Phi_{F(X)} = \Phi_{F(S)} (A_S F_X / A_X F_S) (n_X / n_S)^2$, Where Φ_F is the fluorescence quantum yield, A is the absorbance at the excitation wavelength, F is the area under the corrected emission curve, and n is the refractive index of the solvents used. Subscripts S and X refer to the standard and to the unknown, respectively. The excitation wavelength was at 690 nm while keeping the absorption below 0.05. To calculate the molar extinction coefficient (ε), the absorption at 690 nm of different concentrations of the CTSK-APPA before or after CTSK cleavage (0, 0.5, 1, 2, 5, 10 μM) was measured. According to the Lambert-Beer law, the extinction coefficient was the slope of the fitted curve between concentration and absorption. Both fluorescence quantum yield and extinction coefficient were measured in MES buffer (50 mM, pH 6.0) containing 0.1% DMSO.

***In Vitro* bone-binding and CTSK activation assay.** Hydroxyapatite (HA) was used as a substitute for bone *in vitro* to evaluate the bone-binding ability of CTSK-APPA and CTSK-APP. CTSK-APPA or CTSK-APP (10 μM) was mildly stirred with HA (10 mg/mL) at 37 °C for different times (0, 10, 30, 60, and 120 min). The mixture was centrifugated at 4000 g for 5 min and then washed with a 10-fold excess of PBS (1×) three times. The efficiency of HA binding was determined from the fluorescence intensities of the precipitation according to the equation: $(F - F_{blank})/(F_0 - F_{blank})*100\%$, where F represents the fluorescence intensity of the precipitation incubated with HA for a determined time, and F_{blank} represents the blank fluorescence intensity. After verifying the appropriate bone-binding time, CTSK-APPA or CTSK-APP (10 μM) was mixed with HA (10 mg/mL) at 37 °C for 30 min, and then CTSK (200 ng/mL) was added to the corresponding mixture and incubated at 37 °C for another 1 h under mild stirring. The mixture was centrifugated at 4000 g for 5 min and then washed with a 10-fold excess of MES buffer (50 mM, pH 6.0) three times. Fluorescence images were captured by using the IVIS Spectrum imaging system with excitation at 660 ± 10 nm and emission at 710 ± 10 nm. PA images were captured by using a MSOT scanner with excitation at 700 nm.

Cell culture. Human breast cancer MDA-MB-231/Luc cells were cultured in Leibovitz's L-15 supplemented with 10% FBS and 1% PS in humidified air at 37 °C. Murine macrophage RAW 264.7 cells were cultured in DMEM supplemented with 10% FBS and 1% PS. Bone marrow-derived macrophages (BMMs) were cultured in MEM αsupplemented with 10% FBS and 1%

PS. RAW 264.7 cells and BMMs were maintained in an atmosphere of 5% CO_2 and 95% humidified air at 37 °C. The medium was changed every day.

Cell cytotoxicity assay. MDA-MB-231/Luc and RAW 264.7 cells were seeded in 96-well plates (5×10^3 cells per well) for 24 h and then incubated with CTSK-APPA or CTSK-APP (5, 10, 20, 50 μM) or saline for 24 h. The medium was removed and cells were incubated with CCK-8 (0.5 mg/mL) for another 1 h. The absorbance of the mixture at 450 nm was measured by using a microplate reader. Cell viability was expressed by the ratio of the absorbance of the cells incubated with the probes to that of the cells incubated with culture medium only.

***In vitro* osteoclast differentiation.** Bone marrow-derived macrophages were isolated from the femurs and tibias of 4-week-old C57BL/6J mice and cultured in a complete medium containing M-CSF (30 ng/mL) for 4 days. BMMs were harvested to the 24-well plates (1×10^4 per well) and cultured in the complete medium containing M-CSF (30 ng/mL) and RANKL (100 ng/mL) for another 4 days. The osteoclasts were fixed and stained by using acid phosphatase and a leukocyte kit according to the manufacturer's procedures.

***In vitro* bone resorption imaging assay.** The osteoclasts were reseeded on the surface of the calf bone slice in the 96-well plates and incubated in the complete medium containing M-CSF (30 ng/mL) and RANKL (100 ng/mL) for 5 days with a medium change every other day to achieve a bone resorption model. Then, CTSK-APPA or CTSK-APP (5 μM) was added and incubated with the resorbed bone slice and untreated bone slice at 37 °C for 2 h under mild shaking. Then, the bone slice was washed with MES buffer (50 mM, pH 6.0) three times. For the control experiment, the BMMs were collected and reseeded on the calf bone slice in the 96-well plates and incubated in the complete medium for 5 days with a medium change every other day. After that, CTSK-APPA (5 μM) was incubated with the bone slice at 37 °C for 2 h under mild shaking. The bone slice was washed with MES buffer (50 mM, pH 6.0) three times. For the inhibitory experiment, the bone resorption model was treated with CTSK inhibitor 2-CP (50 mM) at 37 °C for 30 min. Then, the mixture was incubated with CTSK-APPA (5 μM) at 37 °C for 2 h. The bone slice was then washed with MES buffer (50 mM, pH 6.0) three times. Fluorescence images were captured by using the IVIS Spectrum imaging system with excitation at 660 ± 10 nm and emission at 710 ± 10 nm.

Animal model. To establish osteolytic metastasis, MDA-MB-231/Luc cells (1×10^6 cells per mouse) were suspended in 20 μL PBS and injected into the left tibia of specific pathogen-free (SPF) grade 4-week-old BALB/c nude mice under anesthesia using isoflurane. Tumors were allowed to grow for 14 days before imaging experiments.

Bone marrow toxicity analysis. The bone marrow toxicity of CTSK-APPA and CTSK-APP was assessed by blood analyses in mice. SPF grade 4-week-old BALB/c nude mice were intravenously injected with CTSK-APPA(50 μM, 200 μL) or saline (200 μL). The blood samples were collected in anticoagulant tubes at 24 h post-injection and stored at 4°C. The red blood count (RBC) and white blood count (WBC) were further measured.

Renal clearance efficiency studies. SPF grade 4-week-old BALB/c nude mice were intravenously injected with CTSK-APPA or CTSK-APP (50 μM, 200 μL) and placed in metabolic cages. The urine was collected at 3, 6, and 24 h post-injection, and then centrifuged at 5000 r.p.m. for 5 min. The supernatants containing excreted probes were quantified by using a UV-vis absorption spectrometer. The renal clearance efficiency (%) = $A_i/A_t*100\%$, where A_i and A_t were denoted as the UV absorbance at 615 nm of the excreted probes and the total injected probes, respectively.

***In vivo* Bioluminescence, DR, CT, and MR imaging.** For bioluminescence (BL) imaging, MDA-MB-231/Luc tumor-bearing mice were given an intraperitoneal injection of D-luciferin at a dosage of 150 mg/kg. Then BL imaging was recorded on an IVIS Spectrum imaging system at 10 min post-injection under anesthesia using isoflurane. All data were analyzed with the Living Image software. DR, CT, and MR imaging were recorded on an IVIS Spectrum imaging system, a single-photon emission computed tomography imaging system, and a preclinical magnetic resonance solution system, respectively.

Real-time tumor imaging. CTSK-APPA or CTSK-APP (50 μM, 200 μL) was injected through the tail vein. Fluorescence and PA images were acquired at 0, 1, 2, 4, 8, 12, and 24 h post-injection. For the inhibitory group, the mice were pretreated with 2-CP (50 mM, 200 μL) for 1 h, and then fluorescence and PA images were also acquired at 0, 1, 2, 4, 8, 12, and 24 h post-injection. Fluorescence images were captured with excitation at 660 ± 10 nm and emission at 710 ± 10 nm by the IVIS Spectrum imaging system. PA images were acquired from a MSOT scanner at 700 nm.

Histology. All tissues were fixed with 4% paraformaldehyde (PFA), dehydrated in ethanol (EtOH) solution, embedded in paraffin and cut into 10-μm sections for H&E staining. Histology sections were then stained with hematoxylin and eosin following standard protocols. Images were captured by using a fluorescence microscope.

Chemical synthesis and characterization.

Scheme S1. Synthetic route of PEG-ALN.

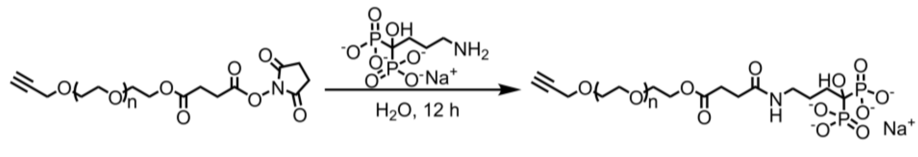

Synthesis of PEG-ALN. PEG-NHS (200 mg, 2 kDa, 0.1 mmol) was slowly added to alendronate sodium aqueous solution (3.3 mg, 1.0 mmol) and the mixture was stirred at room temperature for 12 h. Then, a 10-fold excess of EtOH was added and the mixture was centrifuged at 8000 r.p.m. for 10 min. The supernatant was concentrated under reduced pressure to obtain pure PEG-ALN after lyophilization. ^{1}H NMR of PEG-ALN (D_2O, 400 MHz, Figure S14) δ (ppm): 4.25 (m, 2 H), 4.22 (d, J = 2 Hz, 2 H), 3.89-3.49 (m), 3.19 (t, J = 6 Hz, 2 H), 2.88 (t, J = 2 Hz, 1 H), 2.68 (s, 2 H), 2.55 (t, J = 7 Hz, 2 H), 2.00-1.88 (m, 2 H), 1.85-1.75 (m, 2 H).

Scheme S2. Synthetic route of compound CTSK-APPA and D-CTSK-APPA.

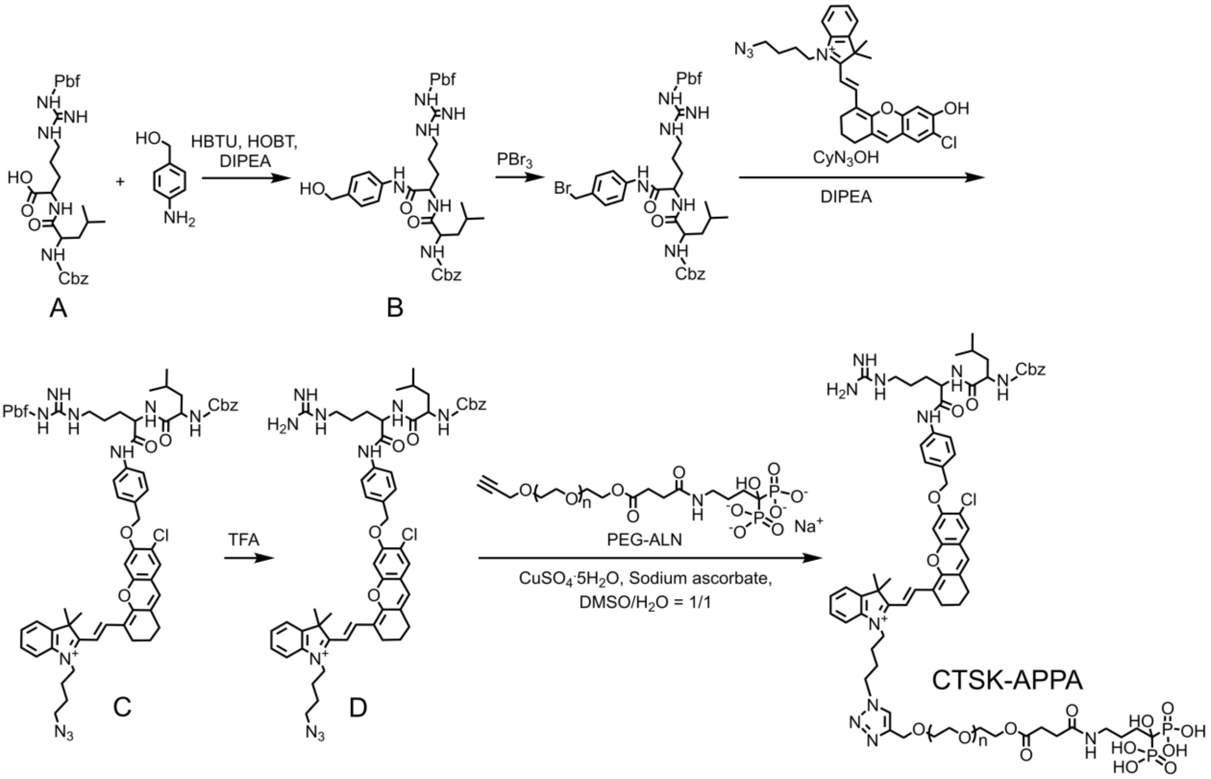

Synthesis of compound A: Compound A (673 mg, 1.0 mmol) was prepared by SPPS. MS of compound A: calculated for $C_{33}H_{47}N_5O_8S$, [$(M+H)^+$]: 674.28; obsvd. MALDI-TOF-MS: m/z 674.20. ^{1}H NMR of compound A ($CDCl_3$, 400 MHz, Figure S15) δ (ppm): 7.50 (d, J = 8 Hz, 2 H), 7.27 (s, 3 H), 5.79 (s, 1 H), 5.06 (d, J = 12 Hz, 2 H), 4.51 (d, J = 5 Hz, 1 H), 4.26 (d, J = 7 Hz, 1 H), 3.25-3,07 (br, 2 H), 2.94 (t, J = 16 Hz, 2 H), 2.50 (s, 3 H), 2.45 (s, 3 H), 2.07 (s, 3 H), 1.87 (m, 1 H), 1.79-1.49 (br, 6 H), 1.46 (s, 6 H), 0.88 (dd, J_1 = 8 Hz, J_2 = 8 Hz, 6 H).

Synthesis of compound B: Compound A (337 mg, 0.5 mmol) was added to a mixture of HBTU (227 mg, 0.6 mmol), HOBT (81 mg, 0.6 mmol) and DIPEA (82 μL, 0.5 mmol) in DMF, and the mixture was stirred at room temperature for 30 min. Then, p-aminobenzyl alcohol (76 mg, 0.55 mmol) was added and the mixture was further stirred at room temperature for another 4 h. The mixture was concentrated under reduced pressure and purified by HPLC to obtain pure product B. MS of compound B: calculated for $C_{40}H_{54}N_6O_8S$, [$(M+Na)^+$]: 801.37; obsvd. MALDI-TOF-MS: m/z 801.48. ^{1}H NMR of compound B ($CDCl_3$, 400 MHz, Figure S16) δ (ppm): 7.49 (d, J = 8 Hz, 2 H), 7.24 (s, 5 H), 7.19 (s, 2 H), 5.97-5.75 (br, 1 H), 5.12-4.91 (m, 2 H), 4.56 (s, 3 H), 4.22 (s, 1 H), 3.15 (s, 2 H), 2.99 (s, 2 H), 2.52 (s, 3 H), 2.46 (s, 3 H), 2.06 (s, 3 H), 1.95-1.79 (br, 1 H), 1.78-1.48 (m, 6 H), 1.45 (s, 6 H), 0.91 (s, 6 H).

Synthesis of compound C: Compound B (156 mg, 0.2 mmol) was dissolved in anhydrous THF in an ice bath under N_2 atmosphere and then phosphorus tribromide (PBr_3) (38 μL, 0.4 mmol) dissolved in anhydrous Tetrahydrofuran (THF) was added. The mixture was stirred in an ice bath under N_2 atmosphere for 3 h. After that, the mixture was concentrated under reduced pressure and dissolved in ethyl acetate (EtOAc). The saturated $NaHCO_3$ solution was added to wash the mixture and the organic phase was collected, and dried over Na_2SO_4. The pure intermediate product was obtained by filtering and concentrating under reduced pressure. The pure intermediate product (52 mg, 0.1 mmol) with further purification was added to the solution of CyN_3OH (42 mg, 0.05 mmol) and DIPEA (17 μL, 0.1 mmol) in anhydrous CH_3CN. The reaction mixture was stirred at 55°C under N_2 atmosphere for 4 h. The pure product C was obtained after HPLC purification. MS of compound C: calculated for $C_{69}H_{82}ClN_{10}O_9S$, [M^+]: 1261.57; obsvd. MALDI-TOF-MS: m/z 1261.75. ^{1}H NMR of compound C ($CDCl_3$, 400 MHz, Figure S17) δ (ppm): 8.46 (d, J = 16 Hz, 1 H), 7.86 (d, J = 8 Hz, 1 H), 7.72 (d, J = 8 Hz, 1 H), 7.63-7.44 (m, 2 H), 7.40-7.29 (m, 2 H), 7.25-7.13 (m, 5 H), 7.00 (s, 1 H), 6.65 (s, 1 H), 5.50-5.20 (m, 2 H), 5.11-4.91 (m, 2 H), 4.53 (s, 1 H), 4.33-4.06 (m, 2 H), 3.45 (t, J = 6 Hz, 1 H), 3.24-3.00 (br, 2 H), 2.92 (s, 2 H), 2.55 (t, J = 12 Hz, 3 H), 2.48 (d, J = 8 Hz, 2 H), 2.05 (s, 3 H), 2.01-1.88 (m, 10 H), 1.72-1.69 (br, 3 H), 1.57 (d, J = 6 Hz, 4 H), 1.44 (s, 6 H), 1.31 (m, 2 H), 1.26 (s, 6 H), 0.87 (m, 6 H).

Synthesis of compound D: Compound C (12.6 mg, 0.01 mmol) was stirred in a mixture solution of dichloromethane (DCM)/trifluoroacetic acid (TFA) (4:1, 2 mL) in an ice bath for 12 h. After the reaction, triethylamine (TEA, 0.8 mL) was slowly added to the mixture and stirred for another 10 min. The mixture was concentrated under reduced pressure and purified by HPLC to obtain pure product D. MS of compound D: calculated for $C_{56}H_{66}ClN_{10}O_6$, [HR-MS]:

1009.4855; obsvd. HR-MS (Figure S18): m/z 1009.4855. ^{1}H NMR of compound D (CD_3OD, 400 MHz, Figure S19) δ (ppm): 8.74 (d, J = 16 Hz, 1 H), 7.71 (d, J = 8 Hz, 3 H), 7.61-7.47 (m, 6 H). 7.31-7.18 (m, 7 H), 6.58 (d, J = 16 Hz, 1 H), 5.37 (s, 2 H), 5.06 (s, 2 H), 4.50 (t, J = 4 Hz, 1 H), 4.41 (d, J = 8 Hz, 2 H), 3.45 (d, J = 8 Hz, 2 H), 3.20 (d, J = 8 Hz, 2 H), 2.71 (d, J = 16 Hz, 4 H), 2.10-1.91 (m, 4 H), 1.84 (s, 6 H), 1.79-1.49 (m, 8 H), 1.43 (s, 2 H), 1.33-1.29 (m, 2 H), 0.90 (dd, J_1 = 8 Hz, J_2 = 4 Hz, 6 H)

Synthesis of compound CTSK-APPA: Compound D (10.1 mg, 0.01 mmol), PEG-alendronate (21.2 mg, 0.009 mmol), $CuSO_4 \cdot 5H_2O$ (12.5 mg, 0.05 mmol) and sodium ascorbate (19.8 mg, 0.1 mmol) were dissolved in DMSO/H_2O (1:1, 2 mL). The reaction was stirred at room temperature under N_2 atmosphere for 12 h. After the reaction, the mixture was added to H_2O dropwise and centrifuged at 8000 r.p.m. for 10 min. The supernatant was collected and dissolved in deionized water for dialysis to obtain pure CTSK-APPA. ^{1}H NMR of CTSK-APPA (CD_3OD, 400 MHz, Figure S20) δ (ppm): 8.68 (br, 1 H), 8.12 (s, 1 H), 7.92-7.04 (br, 16 H), 6.54 (br, 1 H), 5.37 (br, 2 H), 5.05 (s, 2 H), 4.59-4.33 (m, 7 H), 4.22 (m, 3 H), 4.08 (t, J = 10 Hz, 2 H), 3.91-3.41 (m), 3.26-3.12 (m, 4 H), 2.83-2.67 (m, 4 H), 2.60 (t, J = 17 Hz, 2 H), 2.50 (s, 2 H), 2.06 (s, 2 H), 1.99-1.71 (m, 10 H). 1.70-1.48 (br, 5 H), 1.43-1.29 (m, 6 H), 0.90 (t, J = 7 Hz, 6 H). ^{31}P NMR of CTSK-APPA (D_2O, 400 MHz, Figure S21) δ (ppm): 18.28.

Synthesis of D-compound A: D-Compound A (673 mg, 1.0 mmol) was prepared by SPPS. MS of D-compound A: calculated for $C_{33}H_{47}N_5O_8S$, [$(M+H)^+$]: 674.28; obsvd. MALDI-TOF-MS: m/z 674.45. ^{1}H NMR of D-compound A ($CDCl_3$, 400 MHz, Figure S22) δ (ppm): 7.57 (br, 2 H), 7.25 (s, 3 H), 5.94 (s, 1 H), 5.07 (d, J = 12 Hz, 1 H), 4.97 (d, J = 12 Hz, 1 H), 4.51 (s, 1 H), 4.29 (s, 1 H), 3.25-3,09 (br, 2 H), 2.95 (t, J = 16 Hz, 2 H), 2.50 (s, 3 H), 2.45 (s, 3 H), 2.08 (s, 3 H), 1.96-1.82 (br, 1 H), 1.78-1.52 (br, 6 H), 1.46 (s, 6 H), 0.89 (t, J = 8 Hz, 6 H).

Synthesis of D-compound B: D-Compound A (337 mg, 0.5 mmol) was added to a mixture of HBTU (227 mg, 0.6 mmol), HOBT (81 mg, 0.6 mmol) and DIPEA (82 μL, 0.5 mmol) in DMF, and the mixture was stirred at room temperature for 30 min. Then, p-aminobenzyl alcohol (76 mg, 0.55 mmol) was added and the mixture was further stirred at room temperature for another 4 h. The mixture was concentrated under reduced pressure and purified by HPLC to obtain pure D-product B. MS of D-compound B: calculated for $C_{40}H_{54}N_6O_8S$, [$(M+Na)^+$]: 801.37; obsvd. MALDI-TOF-MS: m/z 801.63. ^{1}H NMR of D-compound B ($CDCl_3$, 400 MHz, Figure S23 δ (ppm): 7.52 (d, J = 8 Hz, 2 H), 7.25 (s, 4 H), 7.20 (d, J = 8 Hz, 2 H), 5.90-5.79 (br, 1 H), 5.08-4.94 (m, 2 H), 4.52 (s, 3 H), 4.21 (s, 1 H), 3.12 (br, 2 H), 2.91 (s, 2 H), 2.53 (s, 3 H), 2.47 (s, 3 H), 2.05 (s, 3 H), 1.97-1.82 (br, 1 H), 1.78-1.47 (m, 6 H), 1.44 (s, 6 H), 0.87 (d, J = 8 Hz, 6 H).

Synthesis of D-compound C: D-Compound B (156 mg, 0.2 mmol) was dissolved in anhydrous THF in an ice bath under N_2 atmosphere and then phosphorus tribromide (PBr_3) (38 μL, 0.4 mmol) dissolved in anhydrous Tetrahydrofuran (THF) was added. The mixture was stirred in an ice bath under N_2 atmosphere for 3 h. After that, the mixture was concentrated under reduced pressure and dissolved in ethyl acetate (EtOAc). The saturated $NaHCO_3$ solution was added to wash the mixture and the organic phase was collected, and dried over Na_2SO_4. The pure intermediate product was obtained by filtering and concentrating under reduced pressure. The pure intermediate product (52 mg, 0.1 mmol) with further purification was added to the solution of CyN_3OH (42 mg, 0.05 mmol) and DIPEA (17 μL, 0.1 mmol) in anhydrous CH_3CN. The reaction mixture was stirred at 55°C under N_2 atmosphere for 4 h. The pure D-product C was obtained after HPLC purification. MS of D-compound C: calculated for $C_{69}H_{82}ClN_{10}O_9S$, [M^+]: 1261.57; obsvd. MALDI-TOF-MS: m/z 1261.90. ^{1}H NMR of D-compound C ($CDCl_3$, 400 MHz, Figure S24 δ (ppm): 8.48 (d, J = 16 Hz, 1 H), 7.92 (s, 1 H), 7.73-7.31 (br, 5 H), 7.25-7.16 (m, 3 H), 6.99 (s, 1 H), 6.65 (s, 1 H), 6.29 (d, J = 16 Hz, 1 H), 5.30 (s, 1 H), 5.10-4.92 (m, 2 H), 4.61-4.46 (br, 1 H), 4.38-4.07 (br, 2 H), 3.60 (s, 2 H), 3.03 (s, 2 H), 2.91 (s, 2 H), 2.67 (s, 1 H),2.55 (s, 2 H), 2.48 (s, 2 H), 2.04 (s, 3 H), 1.98-1.56 (m, 10 H), 1.55-1.46 (br, 4 H), 1.42 (s, 12 H), 1.38-1.30 (m, 3 H), 1.28(d, J = 12 Hz, 3 H), 0.87 (t, J = 4 Hz, 6 H).

Synthesis of D-compound D: D-Compound C (12.6 mg, 0.01 mmol) was stirred in a mixture solution of dichloromethane (DCM)/trifluoroacetic acid (TFA) (4:1, 2 mL) in an ice bath for 12 h. After the reaction, triethylamine (TEA, 0.8 mL) was slowly added to the mixture and stirred for another 10 min. The mixture was concentrated under reduced pressure and purified by HPLC to obtain pure D-product D. MS of D-compound D: calculated for $C_{56}H_{66}ClN_{10}O_6$, [HR-MS]: 1009.4855; obsvd. HRMS: m/z 1009.4856. ^{1}H NMR of compound D (CD_3OD, 400 MHz, Figure S25 δ (ppm): 8.73 (d, J = 12 Hz, 1 H), 7.76-7.70 (m, 3 H), 7.61-7.47 (m, 6 H). 7.22 (s, 7 H), 6.58 (d, J = 16 Hz, 1 H), 5.37 (s, 2 H), 5.03 (d, J = 8 Hz, 2 H), 4.63-4.30 (m, 3 H), 3.45 (d, J = 8 Hz, 2 H), 3.19 (t, J = 8 Hz, 2 H), 2.75 (d, J = 16 Hz, 4 H), 2.18-1.89 (m, 6 H), 1.85 (d, J = 8 Hz, 4 H), 1.77-1.52 (m, 8 H), 1.41-1.20 (s, 2 H), 0.96 (dd, J_1 = 8 Hz, J_2 = 4 Hz, 6 H)

Synthesis of compound D-CTSK-APPA: D-Compound D (10.1 mg, 0.01 mmol), PEG-alendronate (21.2 mg, 0.009 mmol), $CuSO_4 \cdot 5H_2O$ (12.5 mg, 0.05 mmol) and sodium ascorbate (19.8 mg, 0.1 mmol) were dissolved in DMSO/H_2O (1:1, 2 mL). The reaction was stirred at room temperature under N_2 atmosphere for 12 h. After the reaction, the mixture was added to H_2O dropwise and centrifuged at 8000 r.p.m. for 10 min. The supernatant was collected and

dissolved in deionized water for dialysis to obtain pure D-CTSK-APPA. ^{1}H NMR of D-CTSK-APPA (C_2D_6SO/CD_3OD, 400 MHz, Figure S26 δ (ppm): 8.66-8.55 (m, 1 H), 7.90 (s, 1 H), 7.69-7.58 (m, 3 H), 7.54-7.35 (m, 6 H), 7.23-7.05 (m, 7 H), 6.48 (d, J = 16 Hz, 1 H), 5.27 (s, 2 H), 4.97-4.90 (m, 2 H), 4.63-4.21 (m, 7 H), 4.19-4.06 (m, 3 H), 4.03-3.94 (m, 2 H), 3.80-3.26 (m), 3.17-2.83 (m, 7 H), 2.81-2.60 (m, 4 H), 2.38 (s, 2 H), 2.06 (s, 2 H), 1.99-1.67 (m, 10 H). 1.61-1.45 (M, 5 H), 1.34-1.07 (m, 6 H), 085 (dd, J_1 = 8 Hz, J_2 = 8 Hz, 6 H).

Scheme S3. Synthetic route of compound CTSK-APP.

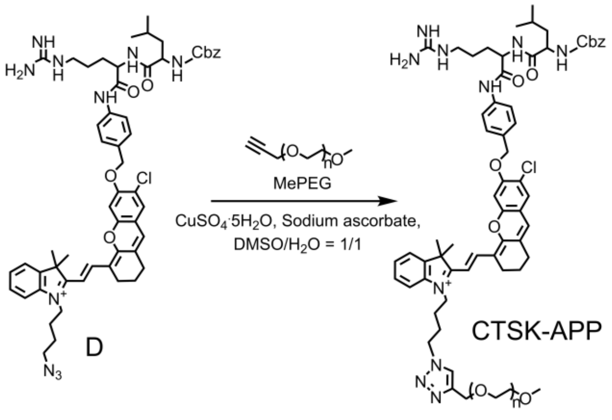

Synthesis of compound CTSK-APP: Compound D (10.1 mg, 0.01 mmol), MePEG (20.0 mg, 0.009 mmol), $CuSO_4 \cdot 5H_2O$ (12.5 mg, 0.05 mmol), and sodium ascorbate (19.8 mg, 0.1 mmol) were dissolved in DMSO/H_2O (1:1, 2 mL). The reaction was stirred at room temperature for 12 h under N_2 atmosphere. After the reaction, the mixture was added into H_2O dropwise and centrifuged at 8000 r.p.m. for 10 min. The supernatant was collected and dissolved in deionized water for dialysis to obtain pure CTSK-APP. ^{1}H NMR of CTSK-APP (CD_3OD, 400 MHz, Figure S27 δ (ppm): 8.64 (d, J = 15 Hz, 1H), 8.06 (s, 1 H), 7.90-7.05 (m, 16 H), 6.49 (d, J = 16 Hz, 1 H), 5.35 (s, 2 H), 5.05 (s, 2 H), 4.67-4.34 (m, 5 H), 4.19 (d, J = 5 Hz, 1H), 4.10 (d, J = 12 Hz, 2 H), 3.99-3.42 (m), 3.36 (s, 3 H), 3.16 (t, J = 10 Hz, 2 H), 2.82-2.58 (m, 4 H), 1.93-1.76 (m, 8 H), 1.71-1.49 (m, 5 H), 1.47-1.22 (m, 6 H), 0.9 (dd, J_1 = 6 Hz, J_2 = 6 Hz, 6 H). MALDI-TOF-MS was shown in Figure S28.

Results and Discussion

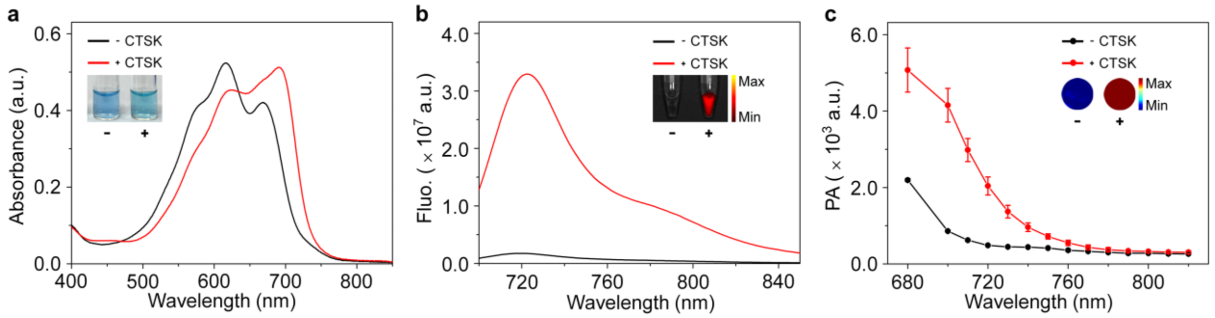

Figure S1. The activation performance of CTSK-APP *in vitro*. UV-Vis absorption (a), fluorescence (Ex = 690 nm) (b), and wavelength-dependent PA signal (c) of CTSK-APP in the absence (-) or presence (+) of CTSK in MES buffer containing 0.1% DMSO (37°C/1 h) (Insets: bright field photographs, fluorescence and PA images of CTSK-APP in Eppendorf tubes recorded before or after treatment with CTSK). All the values are shown as means ± SD (n = 3).

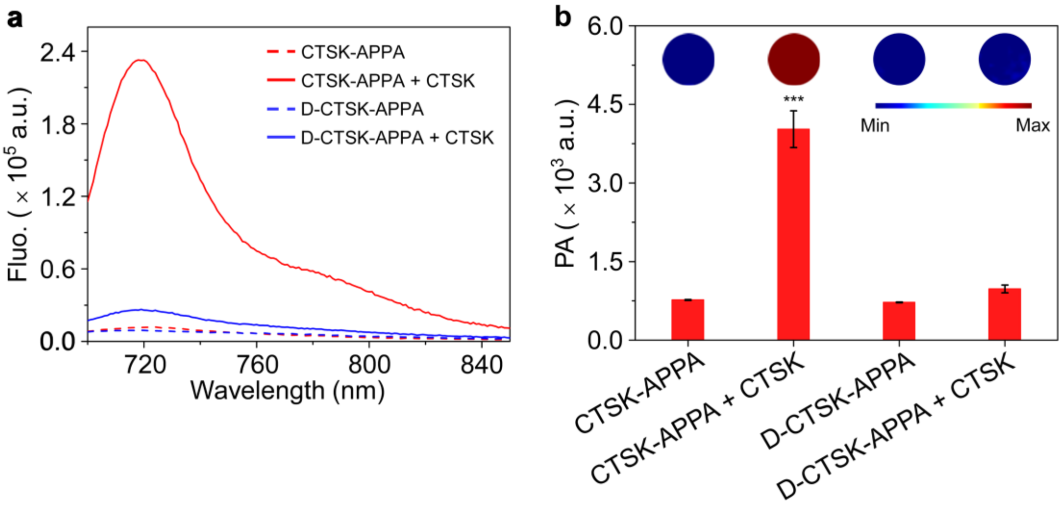

Figure S2. The activation performance of CTSK-APPA and D-CTSK-APPA *in vitro*. fluorescence (Ex = 690 nm) (a) and PA signal (Ex = 700 nm) (b) of CTSK-APPA and D-CTSK-APPA in the absence (-) or presence (+) of CTSK in MES buffer containing 0.1% DMSO (37°C/1 h). All the values are shown as means ± SD (n = 3, one-way ANOVA, ***$p < 0.001$).

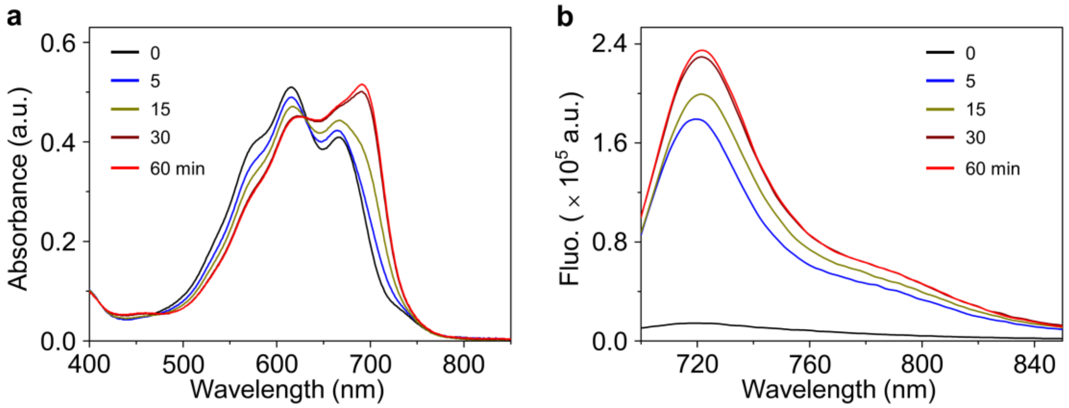

Figure S3. Time-dependent UV-Vis spectra (a) and fluorescence spectra (b) of CTSK-APPA after incubation with CTSK.

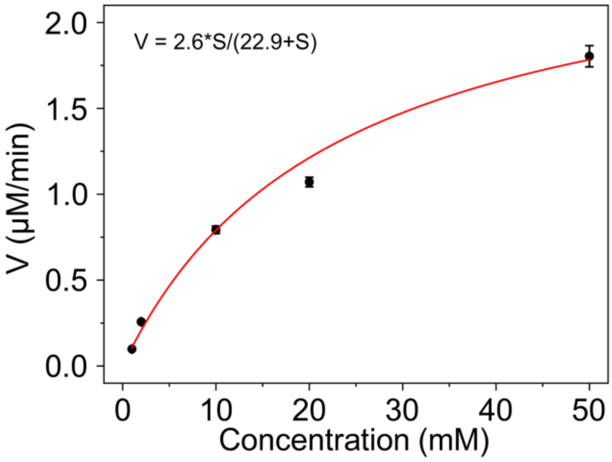

Figure S4. Non-linear regression analysis of CTSK-APPA cleavage rate (V) as a function of CTSK-APPA concentration. All the values are shown as means ± SD (n = 3).

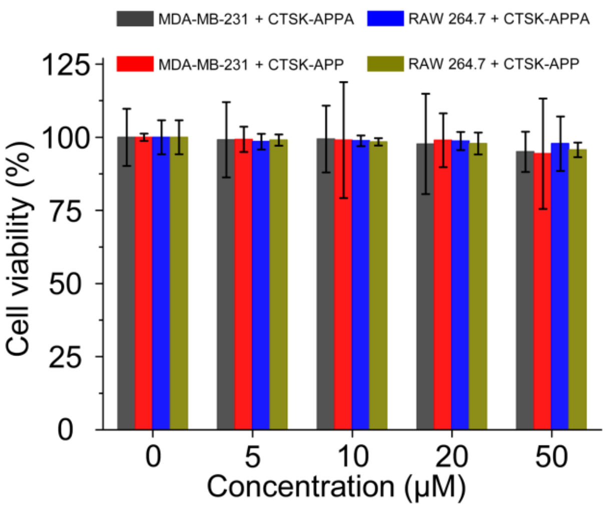

Figure S5. Cell viability of MDA-MB-231 and RAW 264.7 cells treated with different concentrations of CTSK-APPA and CTSK-APP for 24 h. The percentage of viable cells after treatment was calculated relative to cells treated with the same volume of saline. All the values are shown as means ± SD (n = 3).

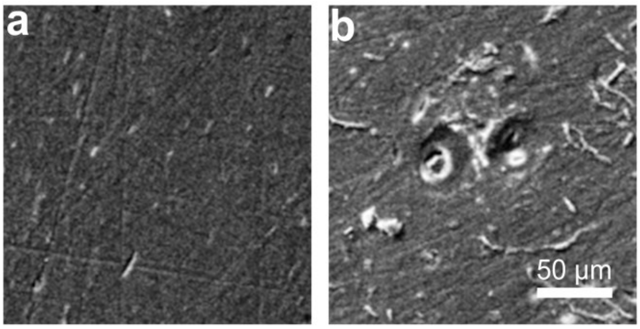

Figure S6. SEM images of the normal bone slices (a) and bone resorption models (b).

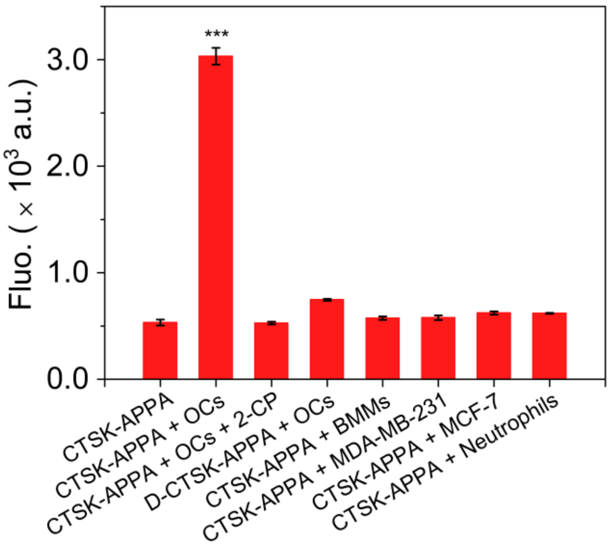

Figure S7. Quantified fluorescence of CTSK-APPA and D-CTSK-APPA after incubation with different cells. All the values are shown as means ± SD (n = 3, one-way ANOVA, ***$p < 0.001$).

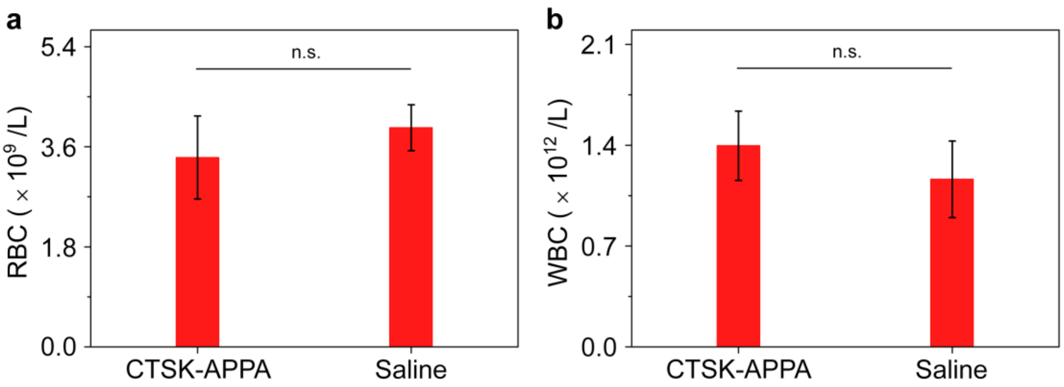

Figure S8. The red blood cell count and white blood cell count of the blood samples were collected from normal mice until 24 h post-intravenous injection of CTSK-APPA. All the values are shown as means ± SD (n = 3, two-sided t-test, n.s.: no statistically significant difference between the two groups).

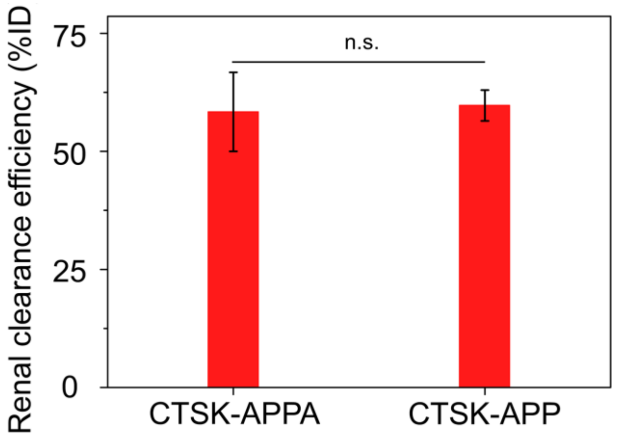

Figure S9. The renal clearance efficiency of CTSK-APPA and CTSK-APP until 24 h post intravenous injection. All the values are shown as means ± SD (n = 3, two-sided t-test, n.s.: no statistically significant difference between the two groups).

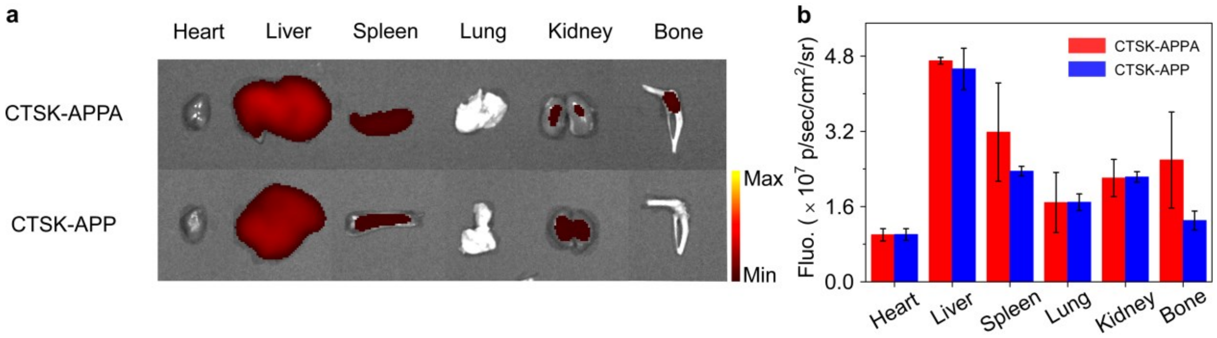

Figure S10. *Ex vivo* fluorescence images (a) and quantification (b) of major organs of mice 24 h after systemic administration of CTSK-APPA or CTSK-APP (n = 3).

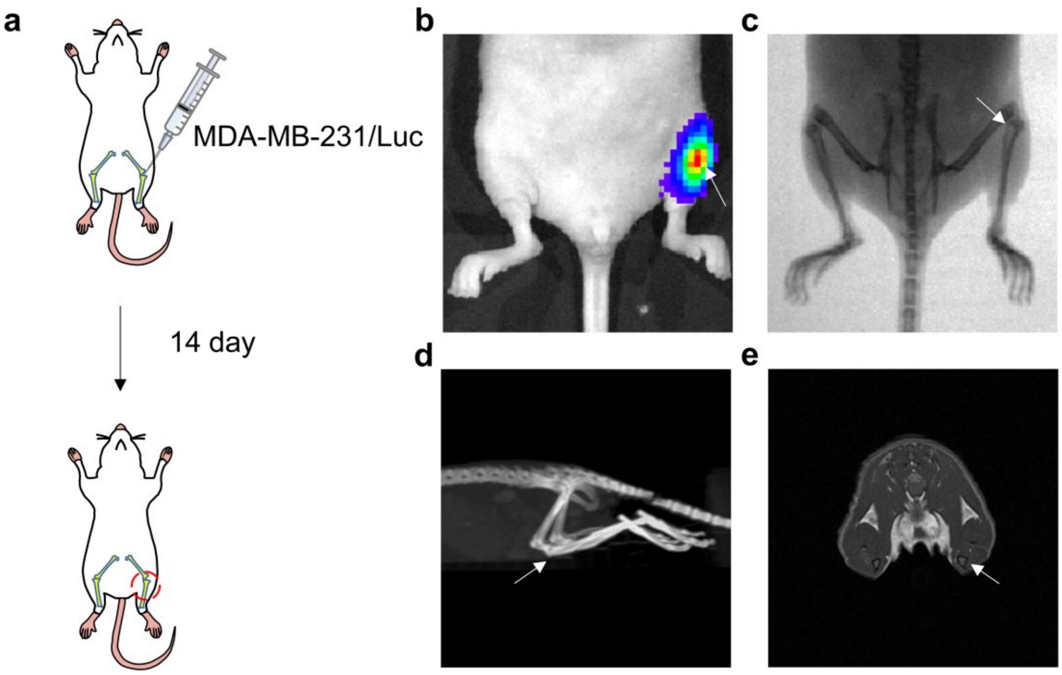

Figure S11. (a) Schematic illustration of the osteolytic metastasis establishment. The images of BL (b), DR (c), CT(d), and MRI (e) are shown the tumor lesion with white arrows.

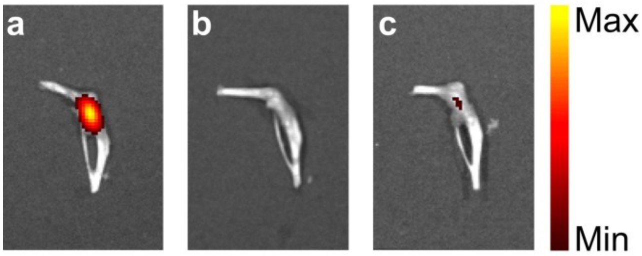

Figure S12. *Ex vivo* fluorescence imaging of bone tissues from sacrificed osteolytic metastasis-bearing mice at 4 h after treatment of CTSK-APPA (a), CTSK-APP (b), and CTSK-APPA together with 2-CP (c).

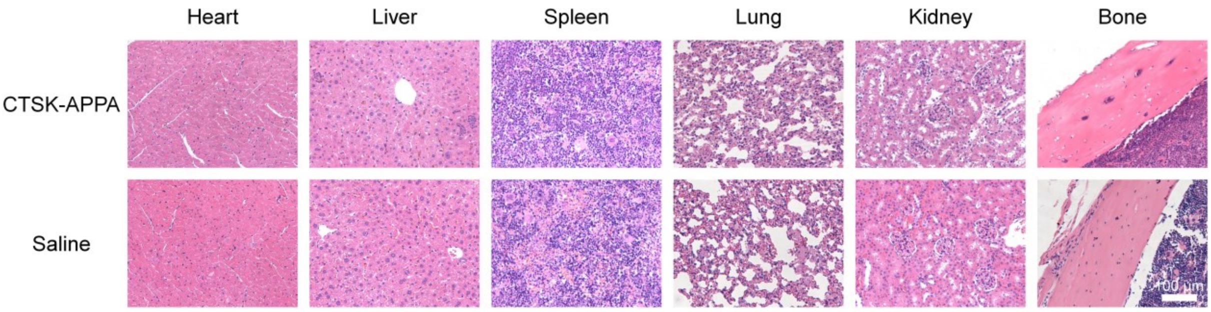

Figure S13. Representative H&E histology of the mice organs until 24 h post intravenous administration of CTSK-APPA or saline. Three separate measurements showed similar results.

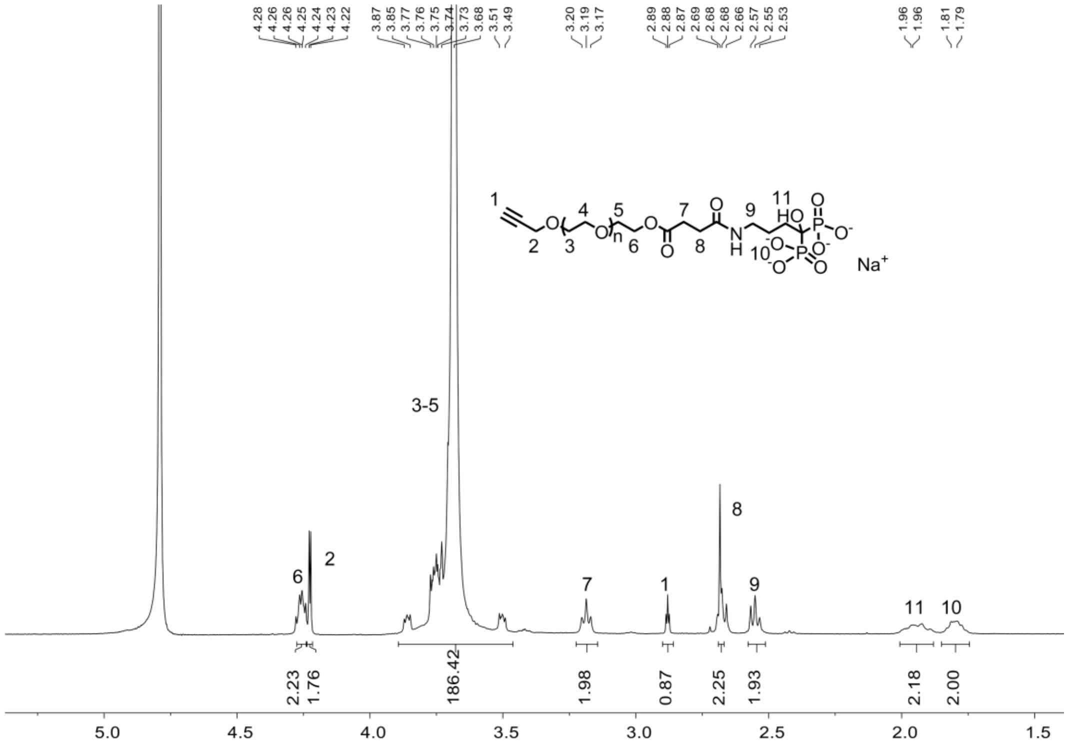

Figure S14. ^{1}H NMR spectrum of PEG-ALN.

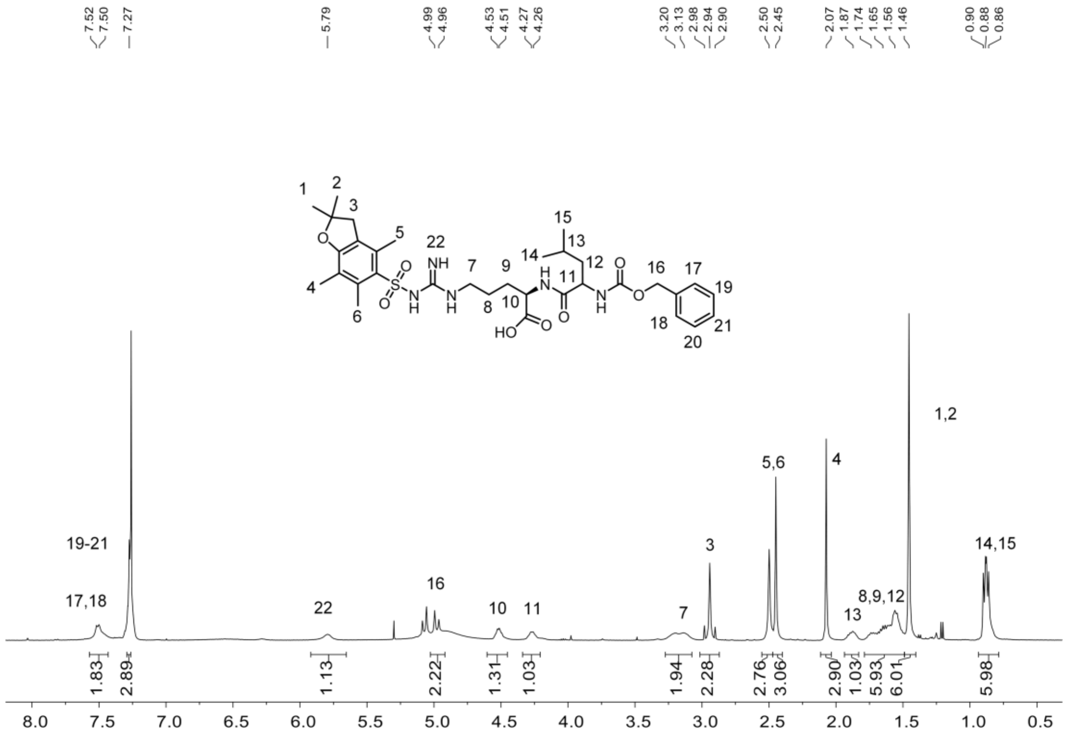

Figure S15. ^{1}H NMR spectrum of compound A.

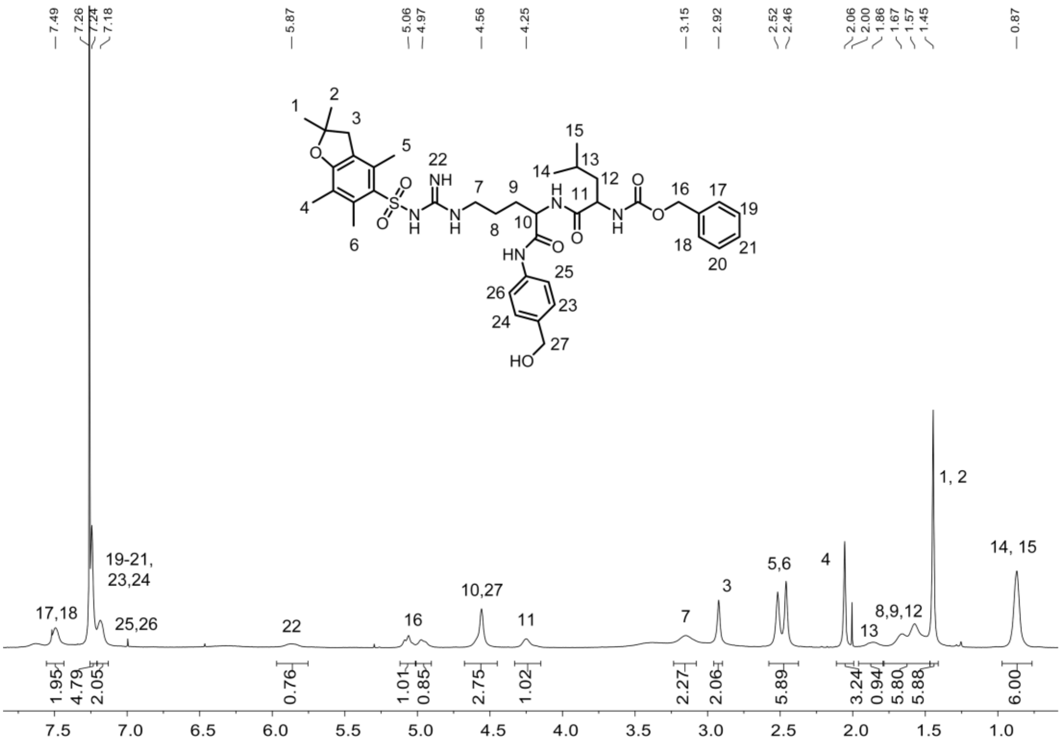

Figure S16. ^{1}H NMR spectrum of compound B.

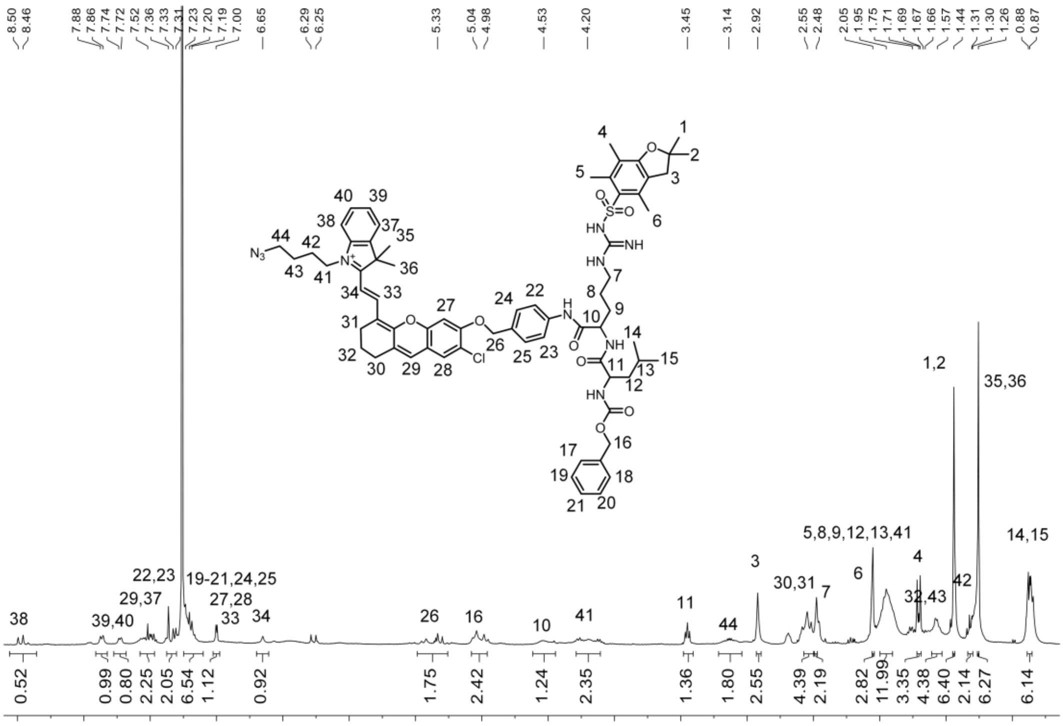

Figure S17. ^{1}H NMR spectrum of compound C.

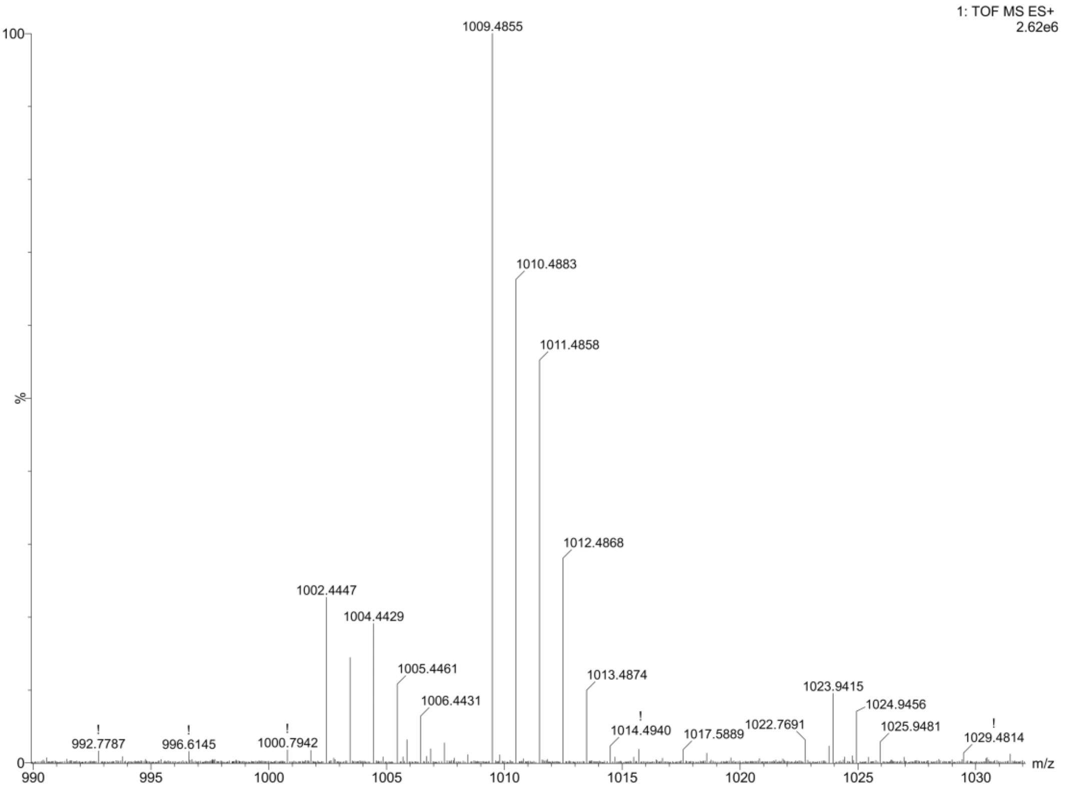

Figure S18. HRMS spectrum of compound D.

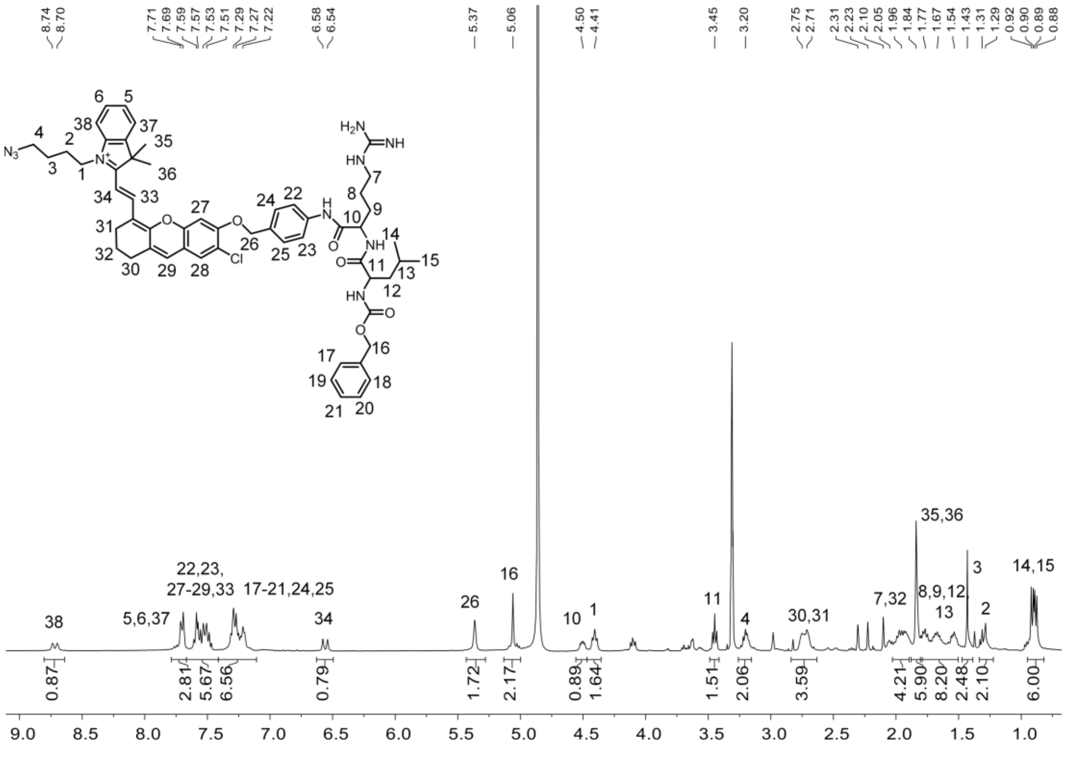

Figure S19. ^{1}H NMR spectrum of compound D.

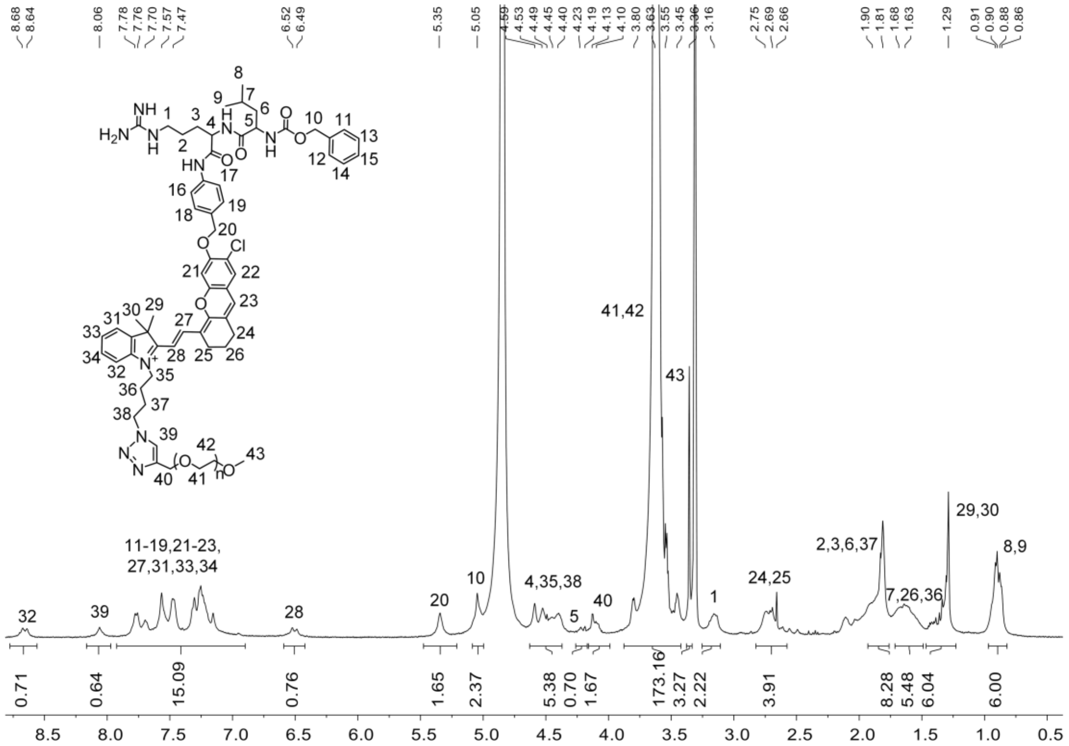

Figure S20. ^{1}H NMR spectrum of CTSK-APPA.

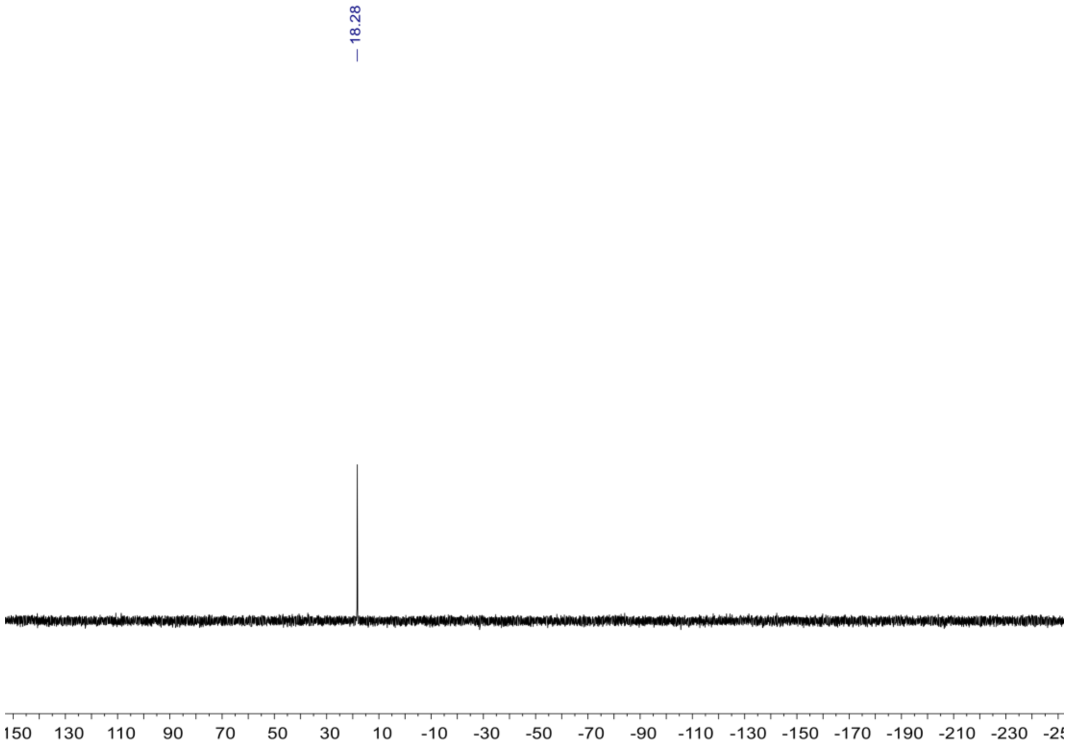

Figure S21. ^{31}P NMR spectrum of CTSK-APPA.

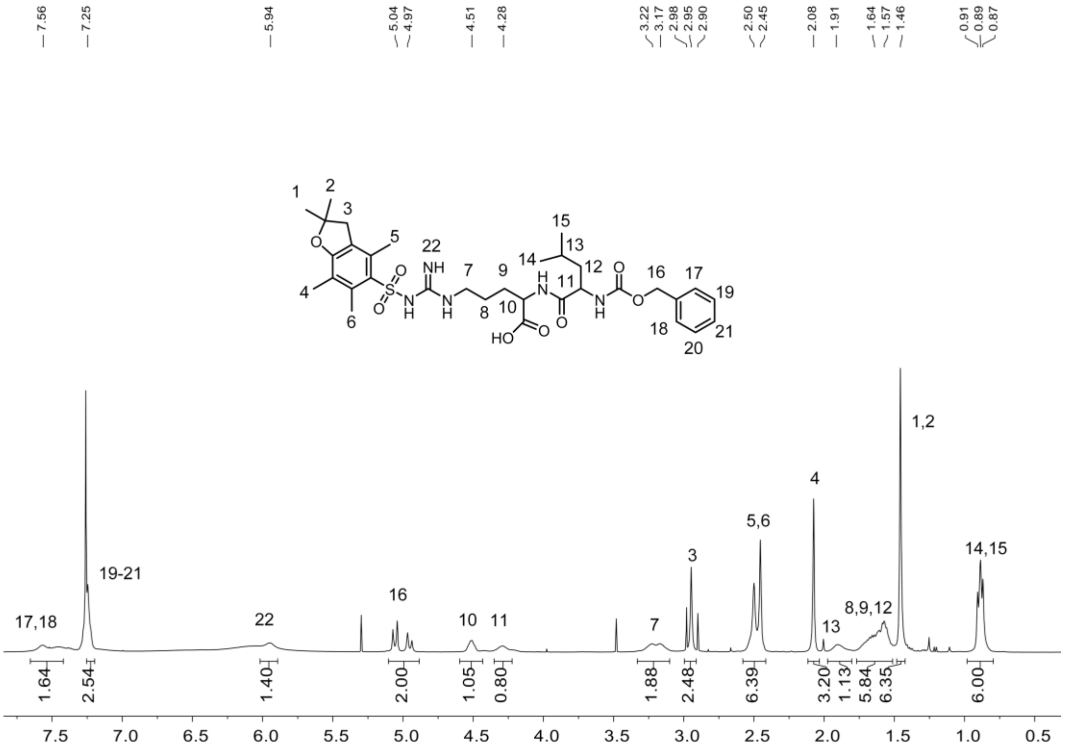

Figure S22. ^{1}H NMR spectrum of D-compound A.

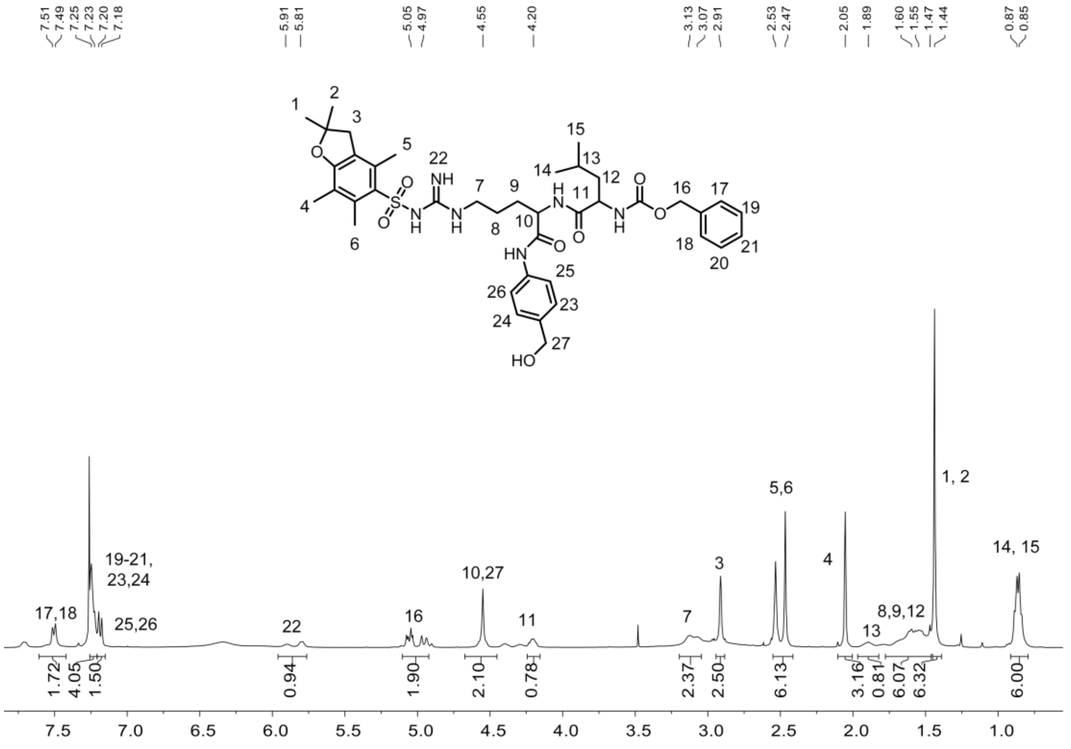

Figure S23. ^{1}H NMR spectrum of D-compound B.

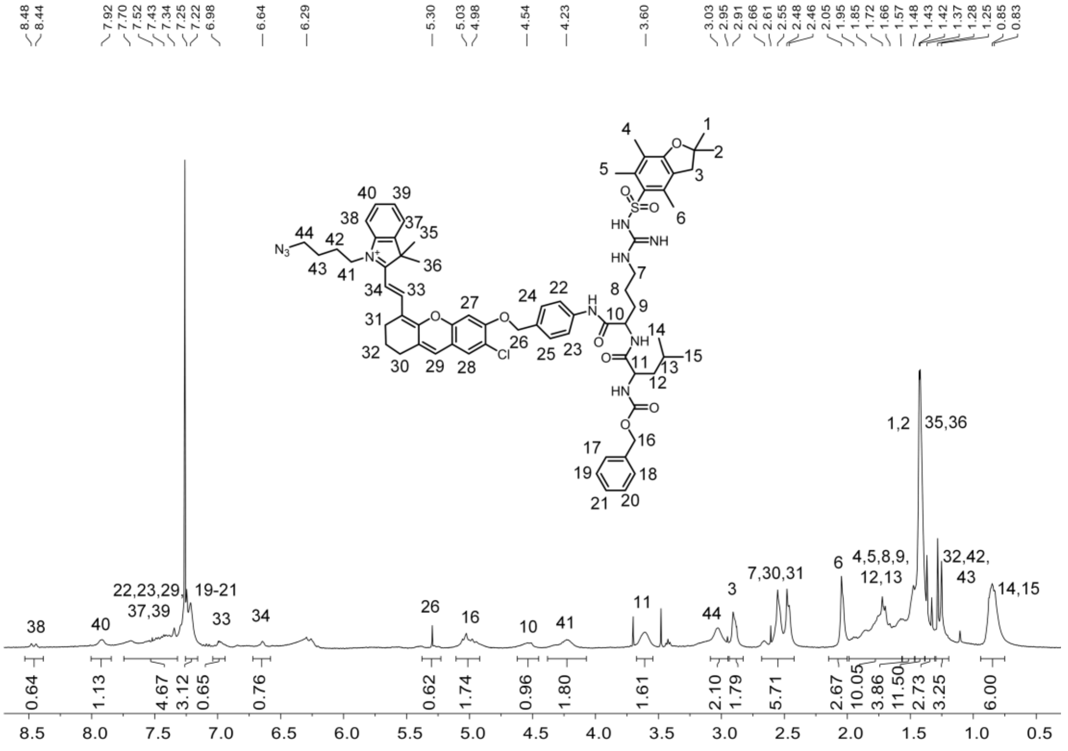

Figure S24. ^{1}H NMR spectrum of D-compound C.

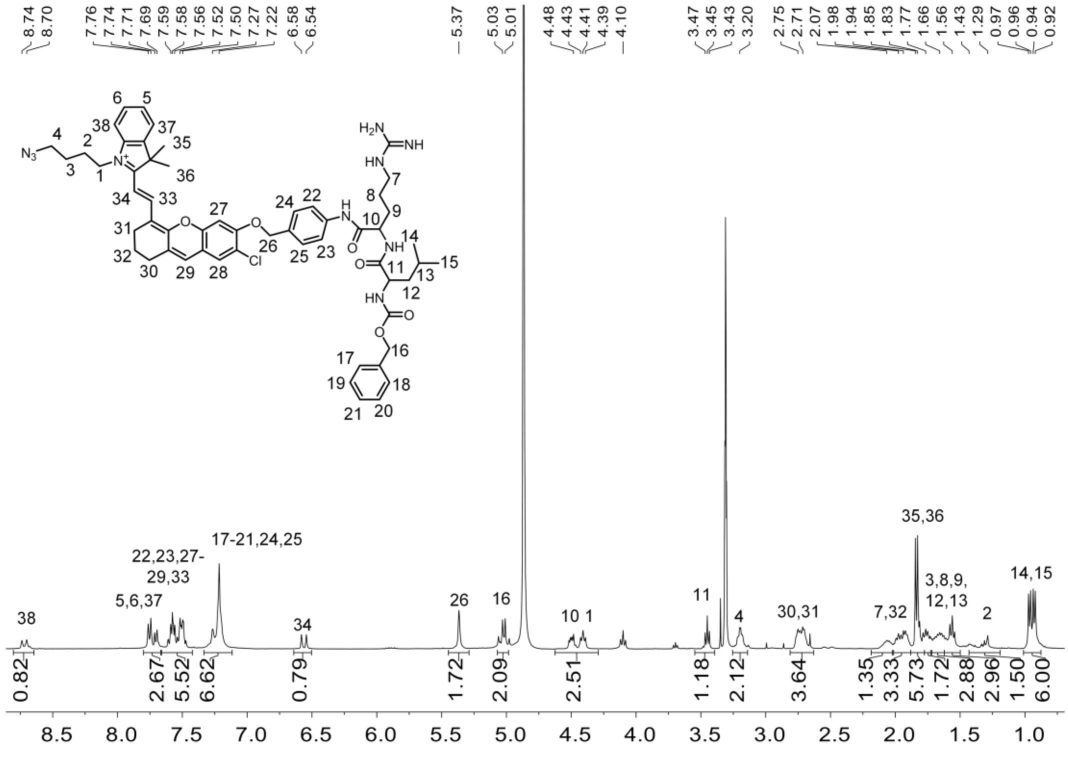

Figure S25. ^{1}H NMR spectrum of D-compound D.

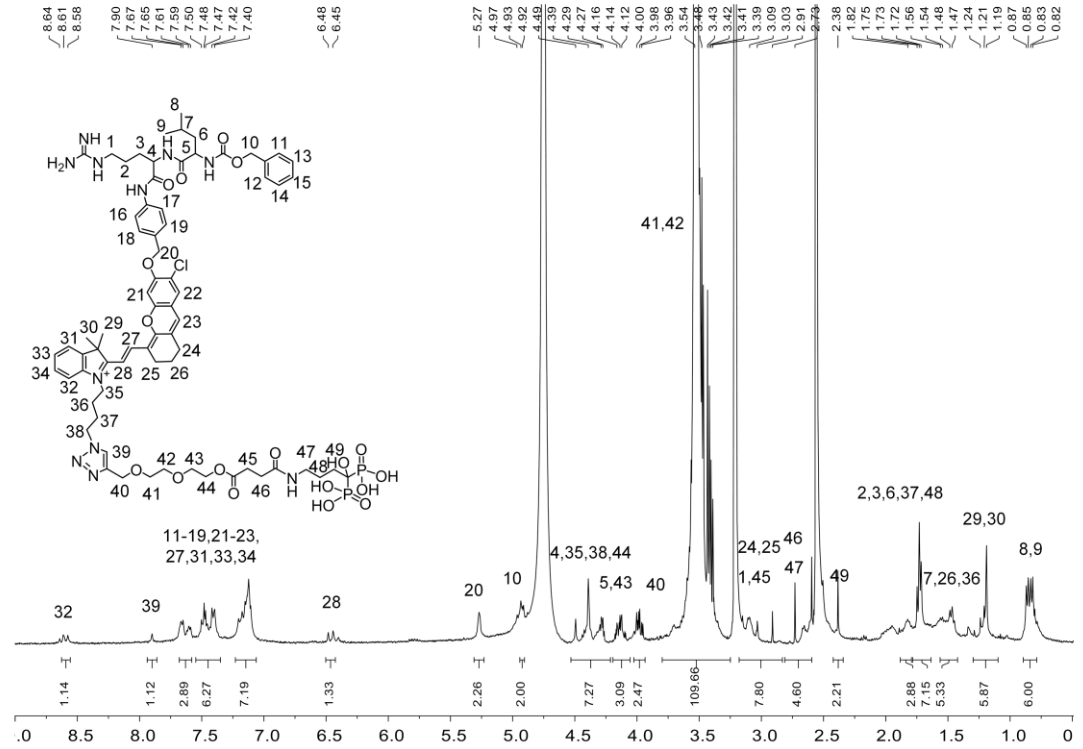

Figure S26. ^{1}H NMR spectrum of D-CTSK-APPA.

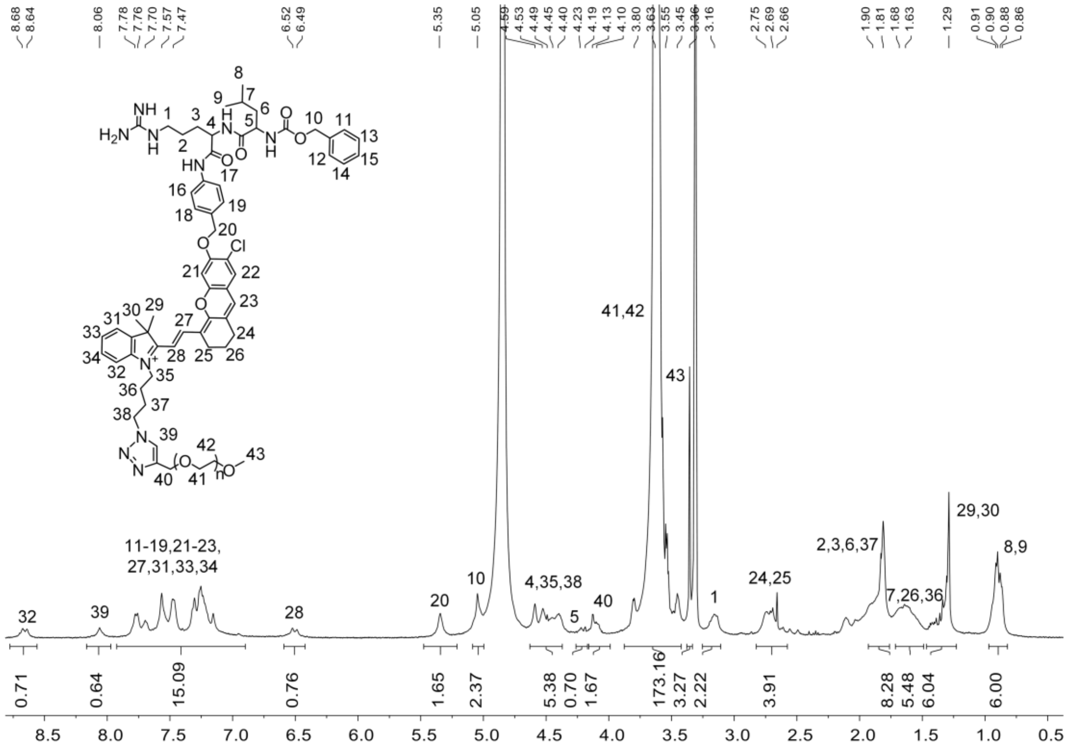

Figure S27. ^{1}H NMR spectrum of CTSK-APP.

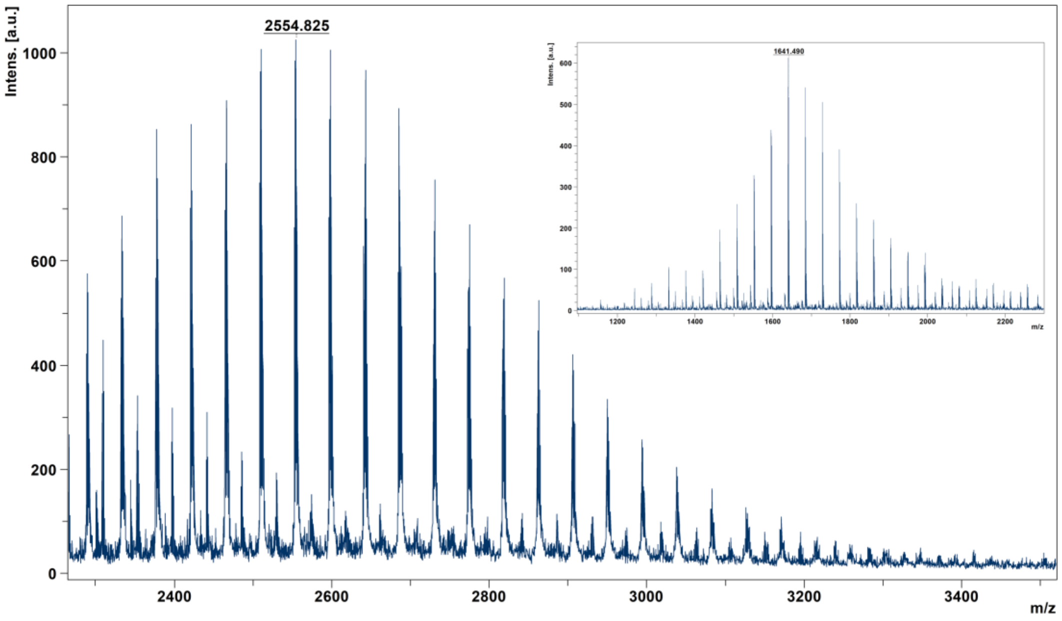

Figure S28. MALDI-TOF-MS spectrum of CTSK-APP, inserted picture: MALDI-TOF-MS spectrum of MePEG for reference.

Table S1. Photophysical data of CTSK-APPA before or after CTSK cleavage in MES buffer (50 mM, pH = 6.0) containing 0.1% DMSO.

	Φ_f (Fluorescence quantum yield)	ε (M^{-1} cm^{-1})
Before cleavage	0.5%	28580
After cleavage	3.5%	50230

Table S2. Properties of CTSK-activatable probes.

Structure	Ex/Em (nm)	k_{cat}/K_m ($M^{-1}s^{-1}$)	Detection limit (ng)	Imaging mode	Reference
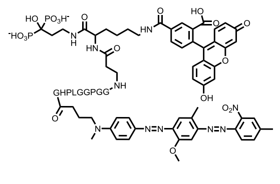	494/518	9.7×10^4	N/A	Fluorescence	[2]

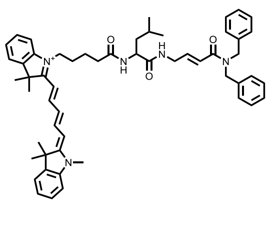	635/660	3.5×10^{4}	25	Fluorescence	[3]
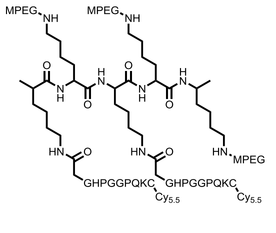	670/720	N/A	N/A	Fluorescence	[4]
	635/695	N/A	N/A	Fluorescence	[5]
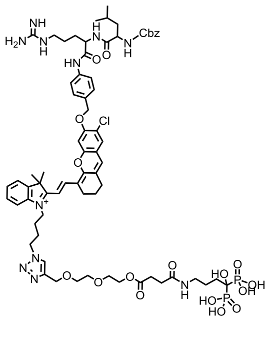	690/720	8.9×10^{5}	0.58	Fluorescence/ photoacoustic	This work

References

[1] Q. Li, S. Li, S. He, W. Chen, P. Cheng, Y. Zhang, Q. Miao, K. Pu, *Angew. Chem. Int. Ed.* **2020**, *59*, 7018-7023.

[2] E. T. Richard, K. Morinaga, Y. Zheng, O. Sundberg, A. Hokugo, K. Hui, Y. Zhou, H. Sasaki, B. A. Kashemirov, I. Nishimura, C. E. McKenna, *Bioconjugate Chem.* **2021**, *32*, 916-927.

[3] C. Lemke, J. Benýšek, D. Brajtenbach, C. Breuer, A. Jílková, M. Horn, M. Buša, L. Ulrychová, A. Illies, K. F. Kubatzky, U. Bartz, M. Mareš, M. Gütschow, *J. Med. Chem.* **2021**, *64*, 13793-13806.

[4] F. A. Jaffer, D. Kim, L. Quinti, C. Tung, E. Aikawa, A. N. Pande, R. H. Kohler, G. Shi, P. Libby, R. Weissleder, *Circulation* **2007**, *115*, 2292-8.

[5] K. M. Kozloff, L. Quinti, S. Patntirapong, P. V. Hauschka, C. H. Tung, R. Weissleder, U. Mahmood, *Bone* **2009**, *44*, 190-198.
